# Biographical analysis of 32 pharmacologists persecuted under the Nazi regime: scientific careers between persecution, emigration, and new beginnings

**DOI:** 10.1007/s00210-025-04231-7

**Published:** 2025-06-09

**Authors:** Mirja Mispagel, Roland Seifert

**Affiliations:** Institute of Pharmacology, Hannover Medical School, Carl-Neuberg-Str. 1, 30625 Hannover, Germany

**Keywords:** National socialism, Third Reich, *Naunyn–Schmiedeberg’s Archives of Pharmacology*, Persecuted pharmacologists, Publication activity, Biographies, *British Journal of Pharmacology*, *Journal of Pharmacology and Experimental Therapeutics*

## Abstract

Founded in 1873, *Naunyn–Schmiedeberg’s Archives of Pharmacology* is the oldest pharmacological journal, and several historical studies have already been published on the occasion of its 150th anniversary. The present study analyzes the influence of the persecution and expulsion of Jewish and dissident German pharmacologists during the Nazi era (1933–1945) on the development of their scientific careers. The analysis is based on the book “*Verfolgte deutschsprachige Pharmakologen 1933–1945*” (engl. “*Persecuted German-speaking pharmacologists 1933–1945*”) by Ullrich Trendelenburg and Konrad Löffelholz (2008), which contains short biographies of 71 persecuted pharmacologists. For this study, 32 of these scientists were selected and their professional development was analyzed in detail. The results show considerable differences in professional establishment after emigration. Young scientists in the early stages of their careers were usually able to successfully establish themselves in their receiving countries and often reached high academic positions, such as professorships or institute directorships. In many cases, established mid-career scientists were also able to continue and develop their careers abroad. On the other hand, senior pharmacologists who had held professorships or directorships before emigrating often found it difficult to obtain comparable positions abroad. Many had to settle for less senior positions. Tragically, two of the pharmacologists analyzed here were murdered in concentration camps. In addition, the biographies and publications of ten pharmacologists—Wilhelm Siegmund Feldberg, Alfred Fröhlich, Hans Kosterlitz, Otto Krayer, Otto Loewi, Hans Molitor, Ernst Peter Pick, Otto Riesser, Klaus Robert Walter Unna, and Marthe Louise Vogt—are analyzed in detail in this study. Their publications in *Naunyn–Schmiedeberg’s Archives of Pharmacology*, the *Journal of Pharmacology and Experimental Therapeutics*, and the *British Journal of Pharmacology* are analyzed in relation to their individual fates and careers. Although there are considerable individual differences, these ten scientists were generally able to successfully establish themselves in their new academic environment despite persecution and emigration. This was mainly due to their exceptional academic commitment, their high level of resilience, and the support they received from international networks and funding organizations. By combining bibliometric and biographical analysis, this study reveals the human and scientific cost of political oppression—and emphasizes the ongoing relevance of protecting academic freedom and diversity in science.

**Supplementary Information:**

The online version contains supplementary material available at 10.1007/s00210-025-04231-7.

## Introduction

On the occasion of the 150th anniversary of the *Naunyn–Schmiedeberg’s Archives of Pharmacology* in 2023, a number of historical articles have been published (Basol and Seifert 2024a, b; Dats et al. 2023; Eyer 2025; Greim 2024a, 2024b; Hattori et al. 2023; Hattori and Seifert 2023; Philippu and Seifert 2023a, 2023b; Toomsalu 2023).

The history of pharmacology in Germany and of the journal *Naunyn–Schmiedeberg’s Archives of Pharmacology* is closely linked to the fate of numerous, mainly Jewish and dissident pharmacologists who were persecuted and marginalized during the Nazi regime in Germany (1933–1945) (Beddies et al. 2014; Grüttner and Kinas 2007; Kröner 1989; Loeffelholz 2011; Medawar and Pyke 2001, Rubin 2014). In a previous paper, we examined the impact of this persecution on the scientific work and publication behavior of German pharmacologists in the journals *Naunyn–Schmiedeberg’s Archives of Pharmacology*, *Journal of Pharmacology and Experimental Therapeutics* (JPET), and *British Journal of Pharmacology* (BJP). Germany suffered an enormous loss of publishing power due to the expulsion of first-class pharmacologists, while the countries of destination of the emigrants benefited greatly from the increase in outstanding scientists (Mispagel and Seifert 2024). While the abovementioned article provides a comprehensive overview of the publications and migration-related changes in the research of the collective under consideration, we are also interested in bringing the individual stories of these scientists into focus.

To ensure a balance between completeness and clarity, we included pharmacologists listed in the book “*Verfolgte deutschsprachige Pharmakologen 1933–1945*” (engl. “*Persecuted German-speaking pharmacologists 1933–1945*”) by Konrad Löffelholz and Ullrich Trendelenburg (2008) who had published in total at least 15 articles in the three journals *Naunyn–Schmiedeberg’s Archives of Pharmacology*, *Journal of Pharmacology and Experimental Therapeutics*, and *British Journal of Pharmacology*, so that we could focus on those with a significant publication output. We analyzed the career trajectories of the 32 German-speaking pharmacologists who met this inclusion criterion. We paid particular attention to the impact of emigration and new beginnings on their scientific careers.

From the group of 32 pharmacologists, we selected the 10 pharmacologists with the most published articles in the three selected journals to analyze their biographies and research in more detail.

A unique aspect of this study is the combination of biographical analysis and bibliometric research, which helps to highlight the individual and structural effects of persecution on scientific publication behavior.

Our previous study showed the far-reaching consequences of the persecution, expulsion, and marginalization of German pharmacologists for the development of pharmacology, particularly in Germany, Great Britain, and the USA. With this study, we wish to make the personal fates behind the figures visible, honor their scientific achievements, and make a contribution to the history of pharmacology.

## Materials and methods

### Analysis of publications in Naunyn–Schmiedeberg’s Archives of Pharmacology, the Journal of Pharmacology and Experimental Therapeutics, and the British Journal of Pharmacology

Established in 1873, *Naunyn–Schmiedeberg’s Archives of Pharmacology* stands as the oldest pharmacological journal (Starke 1998; Dats et al. 2023). Full access to its digital archive is available via SpringerLink (https://link.springer.com/journal/210, last accessed on October 6, 2024). Given that the USA and Great Britain were the primary destinations for many persecuted pharmacologists, we focused on JPET and BJP for comparisons. JPET, published by the *American Society for Pharmacology and Experimental Therapeutics* (ASPET), was founded in 1909 and is recognized as one of the oldest existing journals in the field. BJP, a younger journal, is published by the *British Pharmacological Society* and was established in 1946. Both, JPET and BJP provide online archives (https://jpet.aspetjournals.org/content/by/year, last accessed 20 March 2025; https://bpspubs.onlinelibrary.wiley.com/journal/14765381, last accessed 20 March 2025).

Using the author search, we collected data on all publications by the pharmacologists analyzed here in the three journals mentioned. This included bibliometric information such as authors’ names, article titles, institutional affiliation, submission and publication dates, volume number, number of citations, and digital object identifier (DOI). Charts were created using Microsoft Excel.

### Selection of pharmacologists

This research on persecuted pharmacologists in Germany during the Nazi era was primarily based on the book “*Verfolgte deutschsprachige Pharmakologen 1933–1945*” (engl. “*Persecuted German-speaking pharmacologists 1933–1945*”) by Konrad Löffelholz and Ullrich Trendelenburg (Löffelholz and Trendelenburg 2008), which includes brief biographies of 71 pharmacologists who faced persecution during this period.

Since we could not present the fates of all 71 pharmacologists analyzed in this article, we needed to choose which to focus on. All bibliometric indicators (such as h-index, number of citations, number of publications, impact factor) that attempt to measure the academic influence of a scientist have their specific shortcomings in terms of their informative value (Durieux and Gevenois 2010; Leydesdorff et al. 2016). For our study, we chose the number of articles as the selection criterion for pharmacologists because this metric gives a direct indication of scientific activity and productivity over the period analyzed. This allows us to quantify research output, although we are aware that this alone does not reflect the quality or impact of the work.

**Fig. 1:**
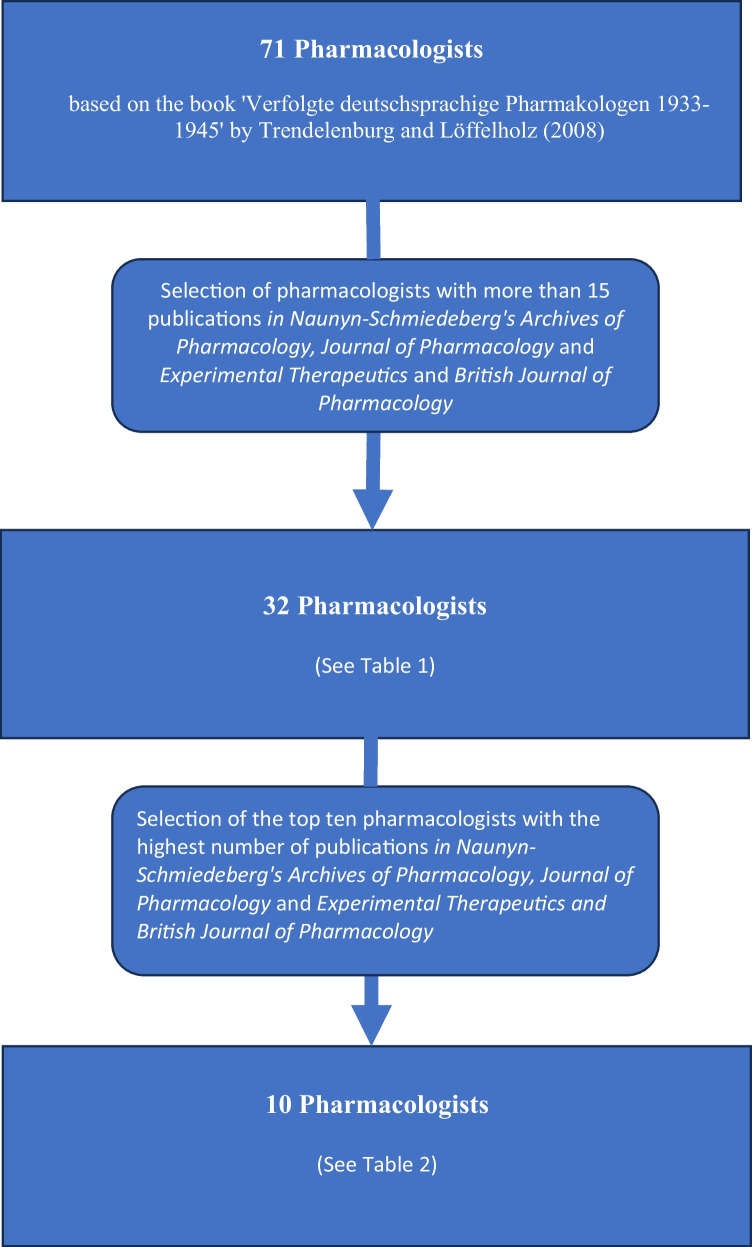
Flow chart showing the selection of pharmacologists analyzed

For the analysis of the persecuted pharmacologists, we defined an inclusion criterion requiring that the scientists in question had published at least 15 articles in *Naunyn–Schmiedeberg’s Archives of Pharmacology*, JPET, and BJP (Table 1). This criterion ensured both a sufficiently large sample of pharmacologists and a clear focus for the study. As a result, a total of 32 pharmacologists were included in our analysis. The selection process can be traced through the flow chart (Fig. 1).

**Table 1:** Selected pharmacologists with more than 15 publications in the pharmacological journals *Naunyn–Schmiedeberg’s Archives of Pharmacology*, JPET, and BJP, listed in alphabetical order of the family name

	Pharmacologist	Number of papers in NSAP	Number of papers in BJP	Number of papers in JPET	Total number of papers in NSAP, BJP, and JPET
1	Blaschko, Hermann Karl Felix	4	17	1	22
2	Born, Gustav Victor Rudolf	-	16	-	16
3	Bueding, Ernst B. (alias Ernest B.)	-	11	13	24
4	Bülbring, Edith	2	11	5	18
5	Dresel, Peter	-	-	17	17
6	Ellinger, Philipp	15	-	-	15
7	Feldberg, Wilhelm Siegmund	13	33	-	46
8	Forst, August Wilhelm	18	-	-	18
9	Freund, Hermann	31	-	-	31
10	Fröhlich, Alfred	39	-	-	39
11	Handovsky, Hans	22	-	-	22
12	Heller, Hans Sigmund	9	5	1	15
13	Kochmann, Martin	18	-	-	18
14	Kohn, Richard (alias Richards, Richard Kohn)	10	1	13	24
15	Kosterlitz, Hans	4	32	2	38
16	Krayer, Otto	19	2	31	52
17	Loewe, Siegfried Walter	20	-	15	35
18	Loewi, Otto	35	-	5	40
19	Meier, Rolf	23	-	-	23
20	Meyer, Hans Horst	21	-	-	21
21	Molitor, Hans	26	-	11	37
22	Peters, Georg (alias Peters, Georges)	33	1	-	34
23	Pick, Ernst Peter	40	-	8	48
24	Pollak, Leo	17	-	-	17
25	Pulewka, Paul	20	-	-	20
26	Riesser, Otto	39	-	-	39
27	Schild, Heinz Otto	2	23	-	25
28	Starkenstein, Emil	29	-	-	29
29	Taubmann, Gert	19	-	-	19
30	Unna, Klaus Robert Walter	9	-	28	37
31	Vogt, Marthe Louise	10	27	-	37
32	Wollenberger, Albert	11	-	7	18

We extracted the birth and death dates, along with the personal histories of the pharmacologists featured in the book. In cases of emigration, we also noted the year and country of emigration as provided in the text.

### Career development of emigrated pharmacologists

To investigate the impact of emigration on the scientific careers of persecuted pharmacologists, we conducted a systematic analysis of their professional positions. Their highest academic positions before and after emigration were identified in order to determine the extent to which it was possible to further develop their careers or return to the academic level of the pre-exile period. We divided the pharmacologists into three groups to analyze the career dynamics in detail: early career scientists (0 to 5 years of experience), mid-career established scientists (6 to 20 years of experience), and advanced or senior scientists (over 20 years of experience). Excel was used to create the graphs.

### “Top-10” persecuted pharmacologists

In order to maintain conciseness and still provide a deeper insight into the fateful biographies of the persecuted pharmacologists, we selected the ten pharmacologists of those under consideration who had published the most papers in *Naunyn–Schmiedeberg’s Archives of Pharmacology*, JPET, and BJP to present their biographies in more detail. The 10 pharmacologists are listed in the alphabetical order of their surnames. We used Excel to create the graphs (Table 2).

**Table 2:** “Top-10” selected pharmacologists and number of publications in the pharmacological journals *Naunyn–Schmiedeberg’s Archives of Pharmacology*, JPET, and BJP

	**Pharmacologist**	**Number of papers in NSAP**	**Number of papers in BJP**	**Number of papers in JPET**	**Total number of papers in NSAP, BJP, and JPET**
1	Feldberg, Wilhelm Siegmund	13	33	-	46
2	Fröhlich, Alfred	39	-	-	39
3	Kosterlitz, Hans	4	32	2	38
4	Krayer, Otto	19	2	31	52
5	Loewi, Otto	35	-	5	40
6	Molitor, Hans	26	-	11	37
7	Pick, Ernst Peter	40	-	8	48
8	Riesser, Otto	39	-	-	39
9	Unna, Klaus Robert Walter	9	-	28	37
10	Vogt, Marthe Louise	10	27	-	37

## Results and discussion

### Analysis of publications in Naunyn–Schmiedeberg’s Archives of Pharmacology, the Journal of Pharmacology and Experimental Therapeutics, and the British Journal of Pharmacology

**Fig. 2:**
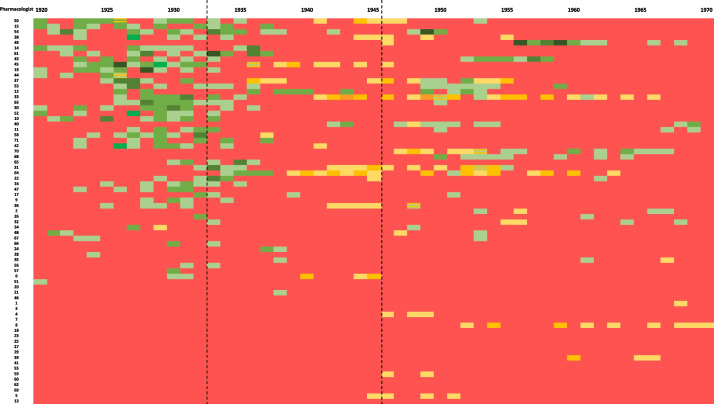
Graph showing the distribution of publications between 1920 and 1970 in NSAP and JPET; the vertical axis shows pharmacologist ID numbers (as listed in Table S1), sorted in descending order by number of publications in NSAP. Higher numbers of publications are positioned at the top. Key to Fig. 2: Color coding of the number of publications per year in the journals *Naunyn–Schmiedeberg’s Archives of Pharmacology* and *Journal of Pharmacology and Experimental Therapeutics* (JPET): no publications in the respective year, red; publications in Naunyn–Schmiedeberg’s Archives of Pharmacology, 1 paper–light green, 2–3 papers–green, 4–5 papers–dark green, > 5 papers–darkest green; publications in JPET, 1 paper–light yellow, 2–3 papers–orange-yellow, 4–5 papers–orange, > 5 papers–brown; publications in both journals in the same year, yellow-green stripes

Our analysis is based on data from the German-language book “*Verfolgte deutschsprachige Pharmakologen 1933–1945*” (engl. “*Persecuted German-speaking pharmacologists 1933–1945*”) by Ullrich Trendelenburg and Konrad Löffelholz (Löffelholz and Trendelenburg 2008), which contains short biographies of 71 persecuted pharmacologists. We have summarized the pharmacologists with dates of birth and death and other biographical data in Table S1 (presented in “Supplements”). We have assigned numbers to the persecuted pharmacologists analyzed here in Table S1, which are also used in Fig. 2.

Figure 2 shows the distribution of all publications of the pharmacologists analyzed here in the pharmacological journals *Naunyn–Schmiedeberg’s Archives of Pharmacology* and JPET. To maintain the aim of the graph, which is to show the development of publications in relation to the Nazi takeover, we have not included publications in the BJP, as it was not founded until 1946, and therefore, the influence of the Nazi takeover in 1933 cannot be shown directly. The green fields show the number of publications of the respective pharmacologist in *Naunyn–Schmiedeberg’s Archives of Pharmacology* in the corresponding year. The darker the shade of green, the more publications were published in that year (see Key to Fig. 2). The same principle applies to the shades of yellow, which indicate the number of publications in each year in JPET. The color coding clearly shows the development of the publication activity of the pharmacologists followed. The high density of publications in the late 1920 s and early 1930 s is followed by a significant decline in publications after the Nazis came to power in 1933. It is also clear that most of the persecuted pharmacologists published for the first time from the late 1930s onwards in the American JPET, which is used here for comparison. This development is directly linked to the persecution, expulsion, marginalization, and emigration of German pharmacologists (Mispagel and Seifert 2024). After the Nazis came to power in 1933, a few publications still appeared in the German journal *Naunyn–Schmiedeberg’s Archives of Pharmacology*, but overall, there was a significant decline in publications by persecuted pharmacologists as a result of the seizure of power.

The subsequent significant increase in the productivity of persecuted pharmacologists after 1940 must also be seen as a sign of their resilience and commitment to the field, as resuming their research activities abroad was a major challenge for many of them (Kröner 1989; Löffelholz and Trendelenburg 2008). Their emigration led to a massive brain drain from Germany (Gerstengarbe 1994; Kohn 2011; Kröner 1989; Mispagel and Seifert 2024; Rall 2017). Among the emigrants were prominent figures such as Otto Krayer, Marthe Louise Vogt, and Nobel Prize Laureate Otto Loewi.

### Career development of emigrated pharmacologists

Emigration from Nazi Germany represented a profound turning point in the scientific careers of the 32 pharmacologists concerned (see Table 1). Many lost their academic positions, their established research environment, and access to funding (Friedländer 2007; Gerstengarbe 1994). At the same time, emigration opened up new professional opportunities for some, who became active in university or industrial research institutions (Kröner 1989; Medawar and Pyke 2001).

To examine the long-term effects of emigration on their academic careers, we analyzed their professional positions before and after emigration.

For better comparability, we divided the pharmacologists studied into three groups:

1. Scientists with 0 to 5 years of professional experience before emigration, who were still in an early career phase.
2. Scientists with 6 to 20 years of experience before emigration, who were already in an established position.
3. Academics with more than 20 years of professional experience before emigrating, who often already held managerial or professorial positions.

This differentiation makes it possible to trace the influence of emigration on different career stages and to analyze whether and to what extent their professional position changed after emigration.

### Scientists with 0 to 5 years of work experience prior to emigration

Eleven of the pharmacologists analyzed here had between none and 5 years of professional experience before emigrating.

These eleven pharmacologists were Gustav Victor Rudolf Born, Ernst B. Bueding (alias Ernest B. Bueding), Edith Bülbring, Peter Dresel, Hans Sigmund Heller, Richard Kohn (alias Richard Kohn Richards), Hans Kosterlitz, Georg Peters (alias Georges Peters), Heinz Otto Schild, Klaus Robert Walter Unna, and Albert Wollenberger (see Table 3).

**Table 3:** Pharmacologists with 0 to 5 years of professional experience before emigration, with their respective year of graduation, age at graduation, age at emigration, number of years of professional experience prior to emigration, and the highest professional position achieved before and after emigration

Pharmacologist	Year graduated	Age at graduation	Age at emigration	Years of employment when emigrating	Pre-migration highest position	Post-migration highest position
Born, Gustav Victor Rudolf	1943	22	12	0	-	Director of Institute
Bueding, Ernst B. (alias Ernest B.)	1933	23	23	0	-	Professor
Dresel, Peter	1952	27	13	0	-	Director of Institute
Peters, Georg (alias Peters, Georges)	1943	23	17	0	-	Professor; Director of Institute
Wollenberger, Albert	1945	33	21	0	-	Professor
Schild, Heinz Otto	1930	24	26	2	Research Associate	Director of Institute
Heller, Hans Sigmund	1931	26	29	3	Research Associate	Professor; Dean of Faculty of Medicine
Unna, Klaus Robert Walter	1930	22	25	3	Research Associate	Professor
Bülbring, Edith	1928	25	30	5	Research Associate	Professor
Kohn, Richard (alias Richards, Richard Kohn)	1930	26	31	5	Research Associate	Professor; Director of Institute
Kosterlitz, Hans	1927	24	30	5	Research Associate	Professor; Director of Institute

**Fig. 3:**
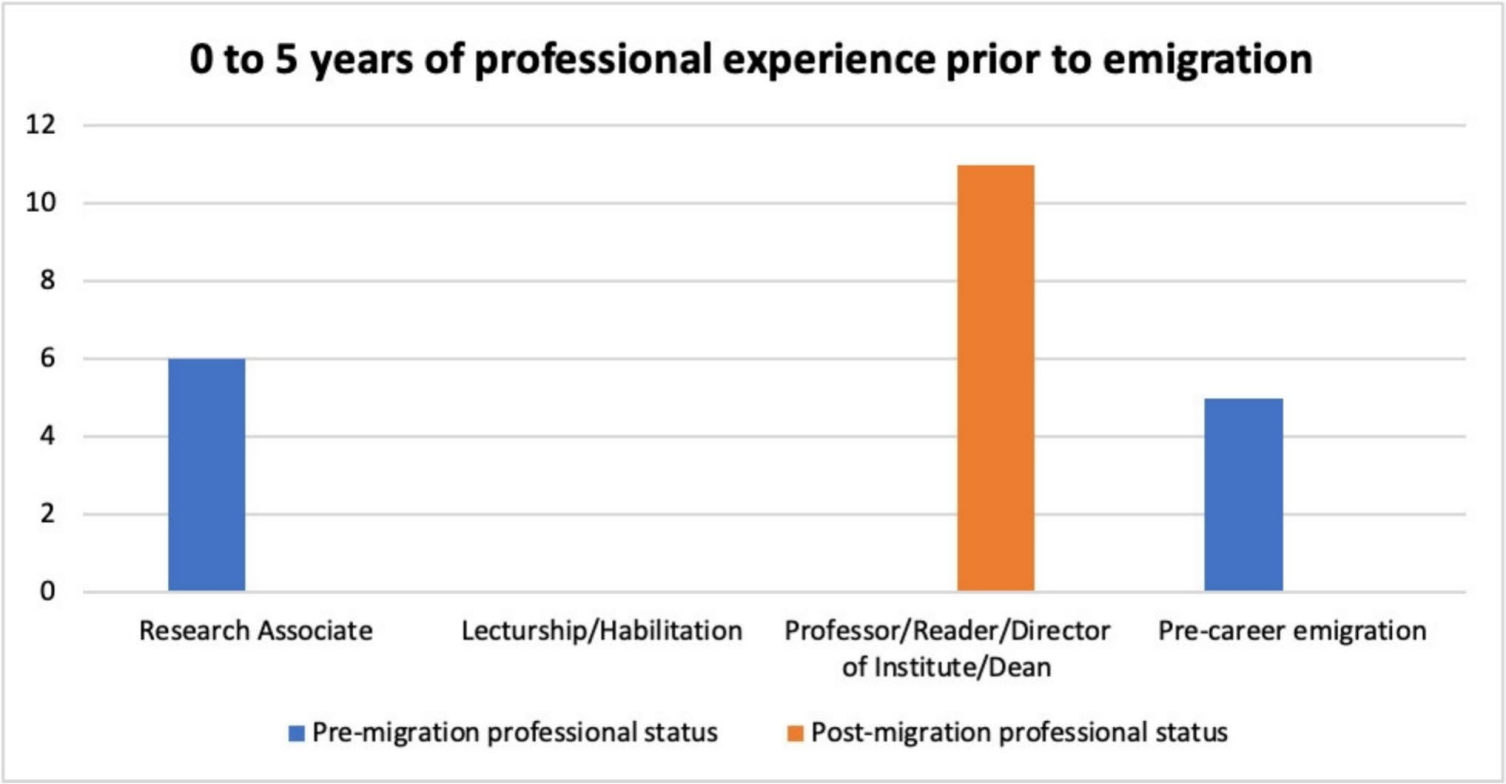
Development of the professional positions of the pharmacologists analyzed here with 0 to 5 years of professional experience prior to emigration; blue, professional positions prior to emigration; orange, professional positions after emigration

Figure 3 shows that five of them emigrated before starting their careers, while the remaining six worked as “research associates.” After emigration, all of these 11 pharmacologists reached top academic positions, becoming professors, readers, and/or directors of institutes. These observations suggest that early emigration had little or no negative impact on their scientific careers, as they were given the opportunity to establish themselves as successful scientists abroad.

A key factor in facilitating this success was the support provided by organizations such as the British *Academic Assistance Council* (AAC), which was established in 1933 to support persecuted academics, particularly those from Germany. The AAC recruited talented academics whose professional development had been restricted by the Nazi regime and provided them with both financial support and access to international academic networks (Boyd et al. 2009; Wordsworth 2019; Brown 2022). These measures enabled many of the emigrants to continue their research abroad and build successful academic careers, often in senior positions (Medawar and Pyke 2001).

It is also likely that their careers were not firmly established in German institutions before they emigrated, which may have facilitated the transition abroad. Less experienced academics may also have been more flexible in their research directions. They may have been less tied to establish working methods or research topics and were therefore able to adapt more easily to new circumstances and topics in their new academic environment.

### Scientists with 6 to 20 years of work experience prior to emigration

Eight of the pharmacologists analyzed here could be classified as established scientists with 6 to 20 years of professional experience. These include Hermann Karl Felix Blaschko, Philipp Ellinger, Wilhelm Siegmund Feldberg, Otto Krayer, Rolf Meier, Hans Molitor, Paul Pulewka, and Marthe Louise Vogt (see Table 4).

**Table 4:** Pharmacologists with 6 to 20 years of professional experience before emigration, with their respective year of graduation, age at graduation, age at emigration, number of years of professional experience prior to emigration, and the highest professional position achieved before and after emigration

Pharmacologist	Year graduated	Age at graduation	Age at emigration	Years of employment when emigrating	Pre-migration highest position	Post-migration highest position
Vogt, Marthe Louise	1929	26	32	6	Research Associate	Lecturship; Professor
Feldberg, Wilhelm Siegmund	1925	25	33	8	Habilitation	Reader; Director of Institute
Krayer, Otto	1925	26	34	8	Professor	Professor; Director of Institute
Blaschko, Hermann Karl Felix	1922	22	33	10	Research Associate	Reader
Molitor, Hans	1921	26	37	11	Professor	Director of Institute
Meier, Rolf	1922	25	38	13	Habilitation	Professor; Director of Institute
Pulewka, Paul	1922	26	39	13	Professor	Professor; Director of Institute
Ellinger, Philipp	1913	26	46	20	Professor; Director of Institute	Research Associate

**Fig. 4:**
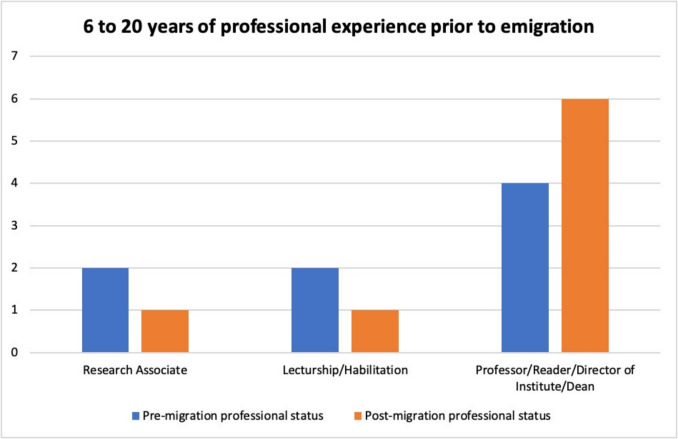
Development of the professional positions of the pharmacologists analyzed here with 6 to 20 years of professional experience prior to emigration; blue, professional positions prior to emigration; orange, professional positions after emigration

Figure 4 shows that also within this group, there was a clear tendency towards professional advancement after emigration. The proportion of academics in leading academic positions—professors, deans, readers, and directors of institutes—increased after emigration compared to before. At the same time, the number of people in lower academic positions decreased.

One exception was the pharmacologist Philipp Ellinger (10 in Table S1). After the “Law for the Restoration of the Professional Civil Service” came into force in 1933, he was dismissed from his professorship at the Pharmacological Institute of the University of Düsseldorf. After emigrating, he did not accept a new professorship, although he was offered an academic position in Ankara. Instead, he chose a different career path. Despite his efforts, he was unable to return to the University of Düsseldorf after the Second World War (Löffelholz and Trendelenburg 2008; Morgan 1953).

Although the graph shows that the majority of the persecuted pharmacologists analyzed here were able to successfully continue their scientific careers despite their forced emigration, it should not be overlooked that this new start was associated with considerable hurdles. In many cases, persecution by the Nazi regime led to considerable uncertainties and career delays (Sziranyi et al. 2019; Uhlendahl et al. 2021; Zeidman et al. 2014, 2017).

At the same time, the professional success of these scientists—despite displacement, discrimination and forced migration—highlights their extraordinary resilience and unwavering commitment to science. Their ability to reorient and develop academically under the most difficult conditions is a testament to their outstanding commitment and scientific excellence.

### Scientists with more than 20 years of work experience prior to emigration

Nine of the pharmacologists analyzed here could be classified as scientists in advanced or leading positions with more than 20 years of professional experience. These include Hermann Freund, Alfred Fröhlich, Hans Handovsky, Siegfried Walter Loewe, Otto Loewi, Ernst Peter Pick, Leo Pollak, Otto Riesser, and Emil Starkenstein (see Table 5).

**Table 5:** Pharmacologists with more than 20 years of professional experience before emigration, with their respective year of graduation, age at graduation, age at emigration, number of years of professional experience prior to emigration, and the highest professional position achieved before and after emigration

Pharmacologist	Year graduated	Age at graduation	Age at emigration	Years of employment when emigrating	Pre-migration highest position	Post-migration highest position
Handovsky, Hans	1912	24	45	21	Professor	Research Associate
Loewe, Siegfried Walter	1910	26	49	23	Professor; Director of Institute	Professor
Starkenstein, Emil	1909	25	55	30	Professor; Director of Institute; Dean	Research Associate; later murdered in concentration camp
Riesser, Otto	1908	26	57	31	Professor; Director of Institute	Lecturship
Freund, Hermann	1906	24	58	34	Professor; Director of Institute	Research Associate; later murdered in concentration camp
Pollak, Leo	1902	24	61	37	Habilitation	Research Associate
Loewi, Otto	1896	23	65	42	Professor	Professor
Pick, Ernst Peter	1896	24	66	42	Professor; Director of Institute	Professor
Fröhlich, Alfred	1895	24	68	44	Professor	Research Associate

**Fig. 5:**
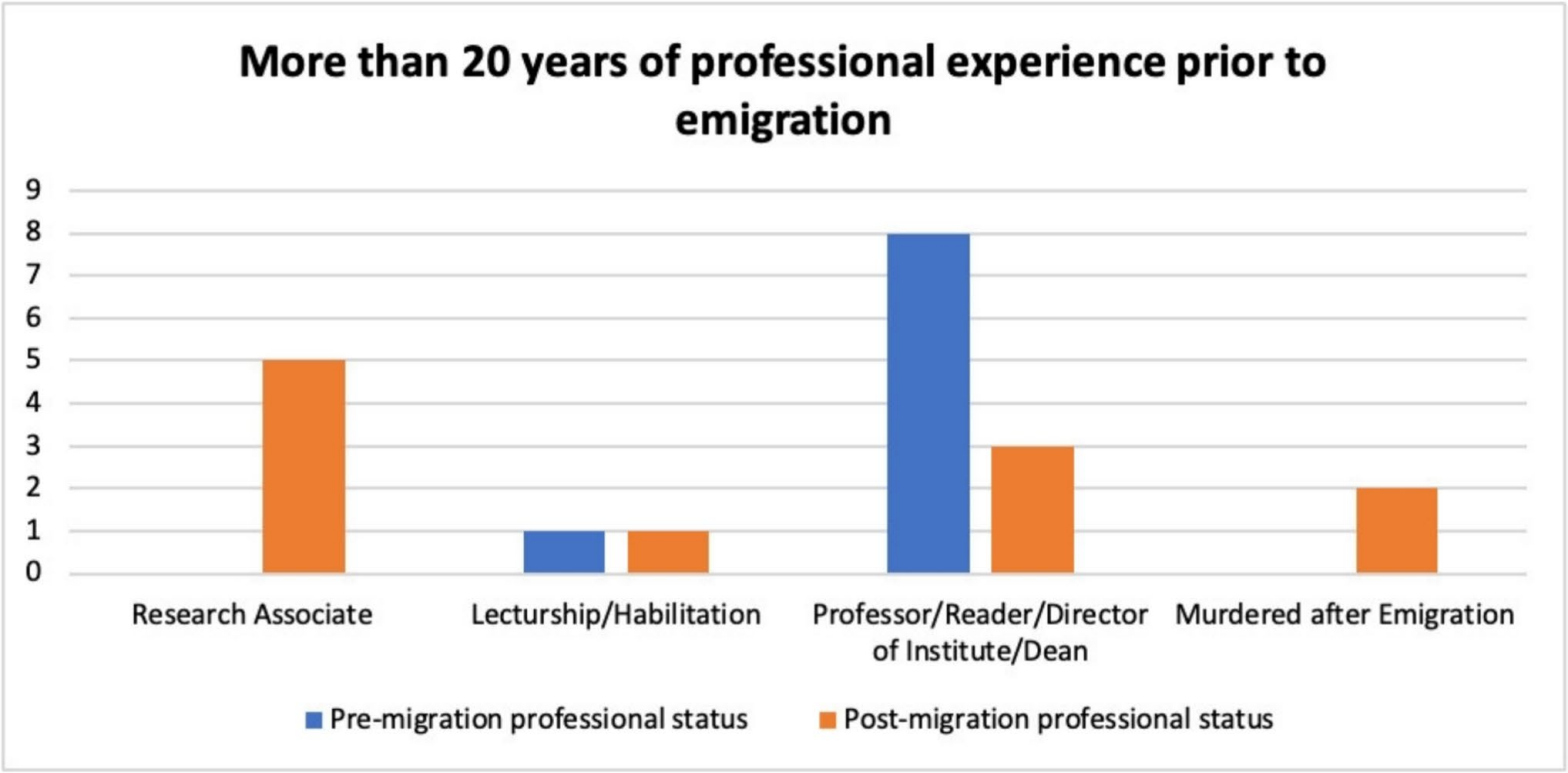
Development of the professional positions of the pharmacologists analyzed here with more than 20 years of professional experience prior to emigration; blue, professional positions prior to emigration; orange, professional positions after emigration

Figure 5 clearly shows that this group was particularly affected by the professional setbacks caused by Nazi discrimination and persecution after emigration. Before emigrating, the majority of them held high-ranking academic positions, such as professors, institute directors, or deans. After emigration, however, only a few were able to regain a comparable academic position: While eight individuals held a professorship or comparable position before emigrating, this number fell to just three after emigrating.

At the same time, the number of academics working as research associates after emigration rose from zero to four. This shows that former leading pharmacologists were in many cases demoted to lower academic positions.

A particularly tragic aspect of this development is that two of the pharmacologists analyzed here were murdered in the course of their careers. These were the pharmacologists Hermann Freund (14 in Table S1) and Emil Starkenstein (61 in Table S1).

Hermann Freund, who had been Professor of Pharmacology and Director of the Institute of Pharmacology at the University of Münster since 1924, was dismissed from his post in 1935 on the basis of the “Reich Citizenship Law of 1935.” In 1939, Freund emigrated to Amsterdam in the Netherlands, where he found employment in a pharmaceutical company. In 1942, however, Freund was arrested by the Gestapo in Amsterdam and deported to Westerbork internment camp in the Netherlands. From there, he was taken to Theresienstadt in 1944 and in the same year to the Auschwitz concentration camp. Hermann Freund was gassed to death in Auschwitz on 14 October 1944 (Huhn and Kilian 2010; Löffelholz and Trendelenburg 2008).

Emil Starkenstein, director and professor of the Pharmacological Institute in Prague from 1929 and dean of the Faculty of Medicine from 1931 to 1932, was dismissed from his post in 1939 after the invasion of the German Wehrmacht on the grounds that he was “non-Aryan.” He then emigrated to the Netherlands, where he found work in a quinine factory in Amsterdam. In 1941, he was taken to Westerbork internment camp, and in 1942, he was deported to Theresienstadt concentration camp. Emil Starkenstein was murdered in the Mauthausen concentration camp on 6 November 1942 (Jezdinský 2006; Junkmann 1947; Löffelholz and Trendelenburg 2008; Senius 1984; Büttner 1989).

These results show that established academics with decades of experience often found it more difficult to re-establish themselves after emigration than their younger colleagues. While younger academics were often still in the early stages of their careers, older academics had already established academic networks and reputations that were often not recognized to the same extent after emigration.

Although some of the scientists analyzed here were able to continue their careers despite these challenges, the graph illustrates that Nazi persecution had a significant and often irreversible impact on the professional lives of these renowned pharmacologists.

### Biographies and publication activity of the “top-10” persecuted pharmacologists

In the following, we analyze the biographies of persecuted pharmacologists and examine their publication patterns in relation to their life trajectories. Our aim is to illustrate the impact of persecution and emigration on their careers and personal lives. For a detailed analysis of publication patterns and biographies, we selected the ten persecuted pharmacologists with the highest number of publications in *Naunyn–Schmiedeberg’s Archives of Pharmacology,* JPET, and BJP (see Table 2).

#### Wilhelm Siegmund Feldberg

Wilhelm Siegmund Feldberg was born on 19 November 1900 in Hamburg. From 1918 to 1925, he studied medicine in Heidelberg, Munich, and Berlin, gaining his doctorate in Berlin in 1925. After graduating, he spent 2 years in the UK, first working with John Langley in Cambridge and then with Henry Hallett Dale at the National Institute for Medical Research in Hampstead (Bisset and Bliss 1997; Löffelholz and Trendelenburg 2008).

He returned to Germany in 1927 to work at the Physiological Institute in Berlin, where he qualified as a professor in 1930 (Bisset and Bliss 1997; Löffelholz and Trendelenburg 2008).

In 1933, when the “Law for the Restoration of the Professional Civil Service” came into force, Feldberg was dismissed as a “non-Aryan”, whereupon he was invited by Henry Dale to continue working with him at the National Institute for Medical Research in Hampstead, as he recognized great potential in Feldberg and his research methods (Bisset and Bliss 1997; Jaenicke 2008; Löffelholz and Trendelenburg 2008).

In 1936, Feldberg was awarded a fellowship at the Walter Eliza Hall Institute in Melbourne, Australia. On his return to England in 1938, he was appointed Lecturer and Reader in the Department of Physiology at Cambridge, a post he held until 1949 (Bisset and Bliss 1997; Löffelholz and Trendelenburg 2008).

From 1949 to 1965, he was the Head of the Department of Physiology and Pharmacology and of the *National Institute for Medical Research* (NIMR) in Hampstead, and from 1965 to 1974, he headed the Laboratory of Neuropharmacology at the NIMR, which was set up especially for him. This was closed in 1974, but Feldberg was able to continue his research with funding from the Medical Research Council (Bisset and Bliss 1997; Jaenicke 2008; Löffelholz and Trendelenburg 2008).

His career came to an end in 1990 when his license to use animals was revoked following allegations by two animal rights activists (Bisset and Bliss 1997).

Feldberg’s research focused mainly on the neurotransmitters histamine and acetylcholine. Through his research, he made fundamental contributions to today’s knowledge of important physiological mechanisms and concepts (Bisset and Bliss 1997; Jaenicke 2008).

Feldberg was elected a *Fellow of the Royal Society* in 1947 (Bisset and Bliss 1997; Löffelholz and Trendelenburg 2008). He was awarded the *Schmiedeberg-Plakette* in 1969 and the *Wellcome Gold Medal* of the *British Pharmacological Society* in 1989 (Bisset and Bliss 1997, https://www.bps.ac.uk/getattachment/Membership-awards/Prizes,-awards-and-grants/Our-prizes/Wellcome-Gold-Medal/Wellcome-Gold-Medal.pdf.aspx?lang=en-GB, last accessed 20 March 2025). He also received honorary doctorates from the universities of Freiburg, Berlin, Cologne, Liège, Würzburg, Heidelberg, Bradford, London, Glasgow, and Aberdeen (Bisset and Bliss 1997).

Wilhelm Siegmund Feldberg died in London on 23 October 1993 at the age of 93 (Bisset and Bliss 1997; Jaenicke 2008; Löffelholz and Trendelenburg 2008).

**Fig. 6:**
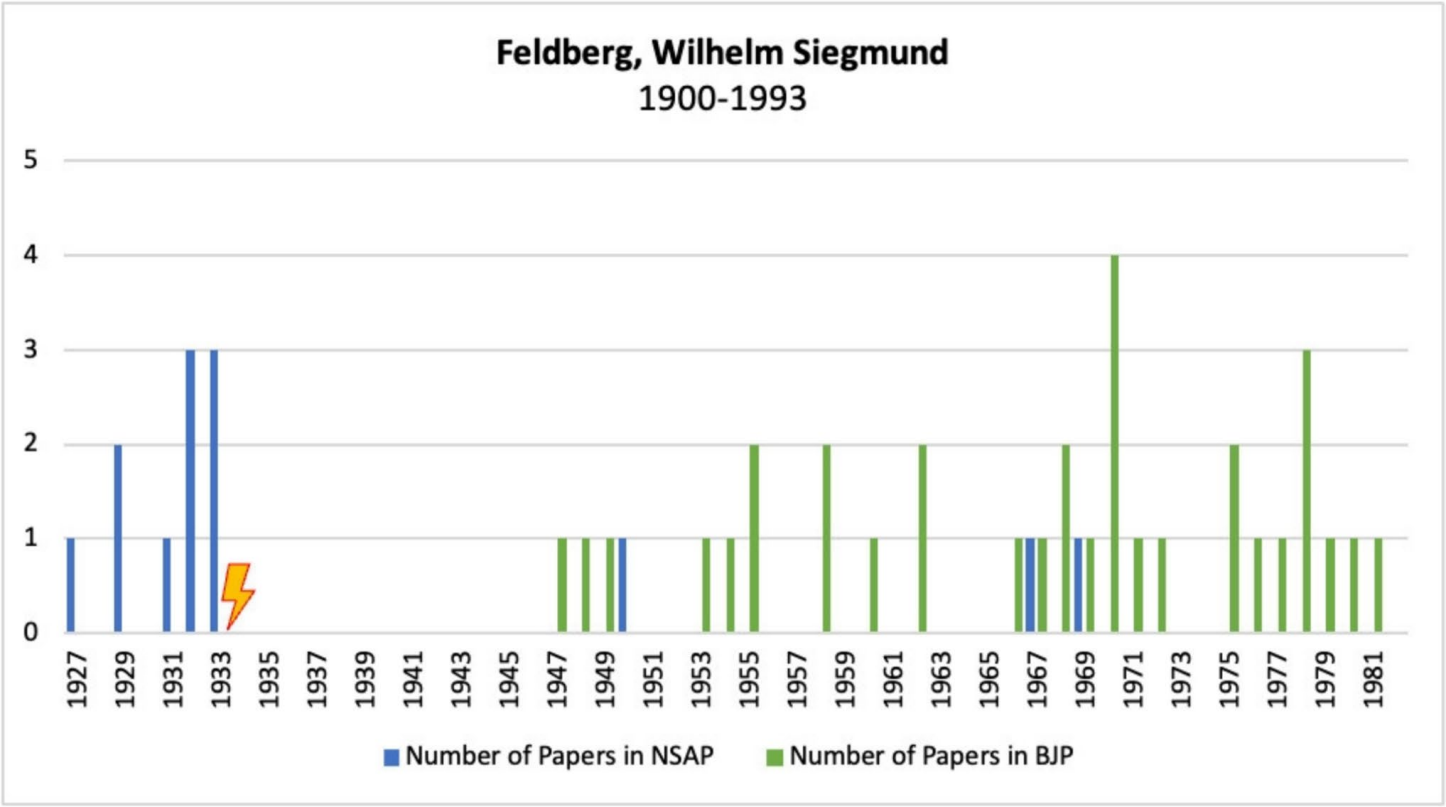
Number of publications by Wilhelm Siegmund Feldberg in *Naunyn–Schmiedeberg’s Archives of Pharmacology*, JPET, and BJP; the lightning bolt symbolizes emigration

Figure 6 shows the publications of Wilhelm Siegmund Feldberg in the journals *Naunyn–Schmiedeberg’s Archives of Pharmacology* and BJP.

Feldberg published several papers in the German journal before his dismissal and emigration in 1933, but no papers appeared for a long time after his emigration. It was not until 14 years later, after the British journal was founded in 1946, that articles by Feldberg appeared in this journal. He continued to publish regularly in the *British Journal of Pharmacology* until 1981.

This pattern shows how many valuable potential publications were lost to the German journal as a result of Feldberg’s dismissal and expulsion.

Feldberg was one of the first expelled pharmacologists to attempt to bring German science and research back into the international arena (Löffelholz and Trendelenburg 2008). In 1961, he founded the “*Stiftung für den deutsch-englischen wissenschaftlichen Austausch für experimentelle medizinische Forschung*” (English: “Foundation for German-English Scientific Exchange for Experimental Medical Research,” later renamed the “Feldberg Foundation”), which promotes scientific contact between Germany and England in the fields of physiology, pharmacology, and related areas, and which still exists today (https://feldbergfoundation.org, last accessed 20 March 2025).

These efforts are also reflected in the three further articles he published after the war in the German journal *Naunyn–Schmiedeberg’s Archives of Pharmacology*. This makes him one of the few displaced pharmacologists analyzed here who published in the German journal again after their emigration.

#### Alfred Fröhlich

Alfred Fröhlich was born in Vienna on 15 August 1871 and studied medicine at the University of Vienna, where he graduated in 1895 (Löffelholz and Trendelenburg 2008; Pick et al. 1953).

From 1895 to 1901, he worked at the Institute of Experimental Pathology at the Medical University Hospital in Vienna. In 1901, he was the first to describe a benign pituitary tumor, the so-called “Fröhlich syndrome” (also known as “Babinski-Fröhlich syndrome” or “dystrophia adiposogenitalis”) (Fröhlich 1901; Löffelholz and Trendelenburg 2008; Pick et al. 1953).

This was followed by 4 years of scientific work in Great Britain, first at the Department of Physiology in Liverpool (1901–1904) and then at the Department of Physiology in Cambridge (1904–1905) (Löffelholz and Trendelenburg 2008; Pick et al. 1953).

In 1906, he returned to Vienna where he worked with Hans Horst Meyer (44 in Table S1), Ernst Peter Pick (50 in Table S1), and Otto Loewi (38 in Table S1) at the Pharmacological Institute of the University of Vienna. In the same year, he qualified as a lecturer in experimental pathology, and in 1910, he qualified as a professor of pharmacology (Löffelholz and Trendelenburg 2008; Pick et al. 1953).

Fröhlich was appointed associate professor in 1912 and full professor in 1922. He retired in 1936 at the age of 65 (Löffelholz and Trendelenburg 2008; Pick et al. 1953).

In 1939, Fröhlich emigrated to the USA as he had been classified as “non-Aryan” by the Nazis. In the USA, he continued his work at the May Institute of Medical Research at the Jewish Hospital in Cincinnati, Ohio, and at the Department of Pharmacology at the University of Cincinnati until his death (Löffelholz and Trendelenburg 2008; Pick et al. 1953).

After a short illness, Alfred Fröhlich died in Cincinnati on 22 March 1953 (Löffelholz and Trendelenburg 2008; Pick et al. 1953).

**Fig. 7:**
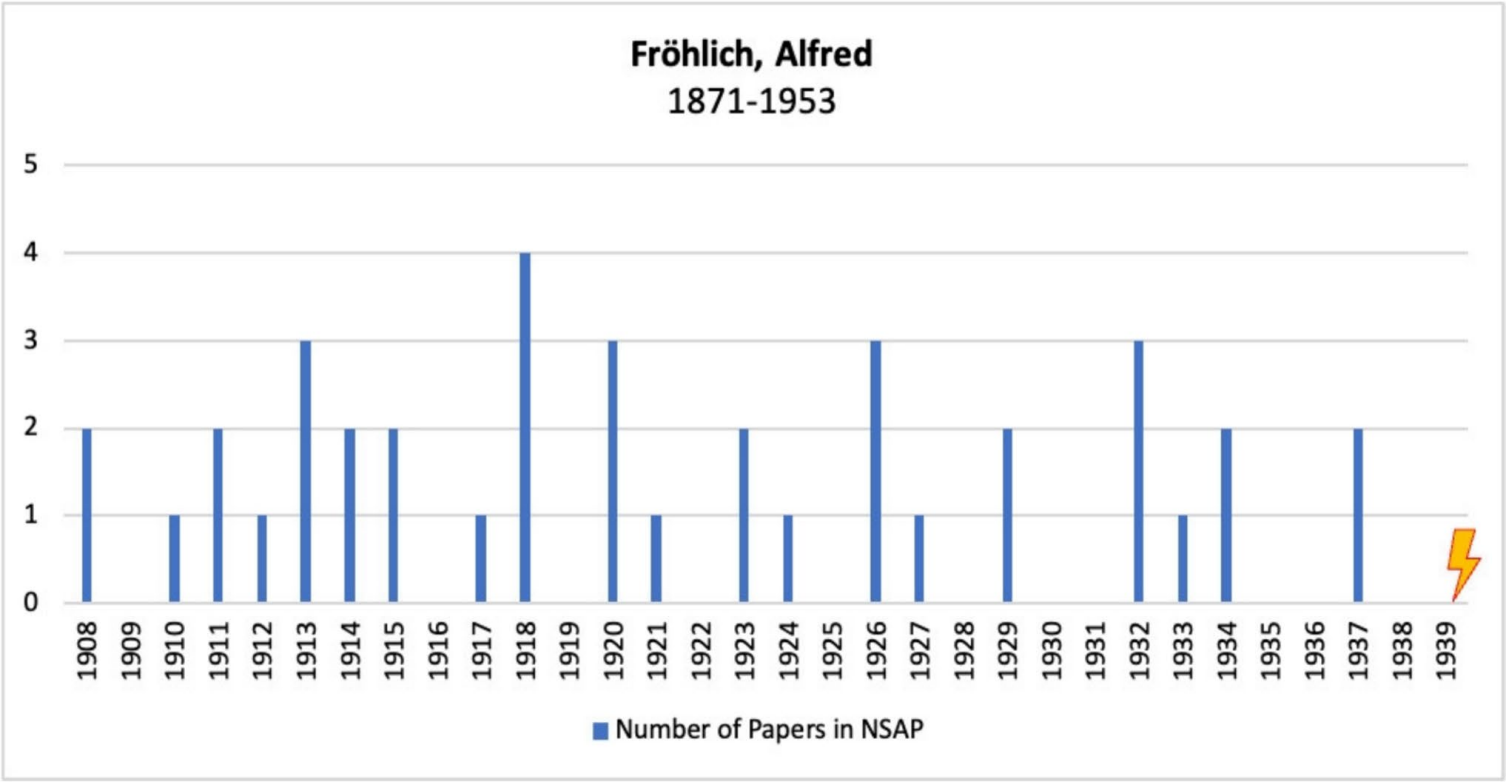
Number of publications by Alfred Fröhlich in *Naunyn–Schmiedeberg’s Archives of Pharmacology*, JPET, and BJP; the lightning bolt symbolizes emigration

Figure 7 shows Alfred Fröhlich’s publications in *Naunyn–Schmiedeberg’s Archives of Pharmacolog*y. Fröhlich published a considerable number of articles in this German journal with great consistency before he emigrated. After his emigration, however, his publications in the German journal came to a standstill. Although he emigrated to the USA and remained active there for many years, he did not publish in the American journal analyzed here, JPET. Fröhlich was a highly respected scientist in Germany at the time (Pick et al. 1953; Triarhou 2022, 2023). Although he retired in 1936, he continued to work at the Pharmacological Institute in Vienna until Austria was annexed by the Nazis in 1938 and he finally emigrated in 1939 (Schütz et al. 2018; Triarhou 2023). His career in Germany came to an early end. After emigrating to the USA, Fröhlich was unable to obtain a professorship, although his age of 68 at the time may have played a role.

#### Hans Walter Kosterlitz

Hans Walter Kosterlitz was born in Berlin on 27 April 1903 and studied medicine in Heidelberg, Freiburg im Breisgau and Berlin until 1927. From 1928 to 1933, he worked at the First Medical Clinic in Berlin and was awarded a doctorate in medicine in 1929 (Löffelholz and Trendelenburg 2008; North and Hughes 2013).

In 1933, when the “Law for the Restoration of the Professional Civil Service” came into force, he was dismissed because he was classified as “non-Aryan.” Kosterlitz then emigrated to Great Britain at the age of 30, where he vowed never to speak German again (Hughes, John 1996, Löffelholz and Trendelenburg 2008; North and Hughes 2013).

In Great Britain, he worked from 1934 to 1968 at the Department of Physiology in Aberdeen, where he obtained his doctorate in 1936 and became a lecturer in 1938. He was promoted to D.Sc. in 1944 and Senior Lecturer in 1945 (Hughes, John 1996, Löffelholz and Trendelenburg 2008; North and Hughes 2013).

From 1953 to 1954, he spent a sabbatical year in the Department of Pharmacology at Harvard Medical School, working under the direction of Otto Krayer (33 in Table S1) (Hughes, John 1996, Lees 1998; Löffelholz and Trendelenburg 2008; North and Hughes 2013).

In 1968, Kosterlitz became Director of the Department of Pharmacology at the University of Aberdeen, retiring in 1973 (Hughes, John 1996, Lees 1998; Löffelholz and Trendelenburg 2008; North and Hughes 2013). After his retirement, he continued to work until 1996 at the Unit for Research on Addictive Drugs in Aberdeen, where, together with J. Hughes, he made the discovery of endorphins in 1975 (Hughes, John 1996, Hughes et al. 1975, Lees 1998; Löffelholz and Trendelenburg 2008; North and Hughes 2013).

Kosterlitz received numerous honors, including the Schmiedeberg Medal in 1976, election as a Fellow of the Royal Society in 1978, the Royal Medal of the Royal Society of London in 1979, and honorary membership of the British Pharmacological Society in 1980. He was awarded the Feldberg Prize in 1981 and the Wellcome Gold Medal of the British Pharmacological Society in 1987 (Hughes, John 1996, Lees 1998; Löffelholz and Trendelenburg 2008; North and Hughes 2013).

Kosterlitz’s main areas of research included carbohydrate metabolism, the autonomic nervous system, opiates, and opioids (Hughes, John 1996, Lees 1998; North and Hughes 2013).

Hans Walter Kosterlitz died in Aberdeen on 26 October 1996 at the age of 93 (Hughes, John 1996, Lees 1998; Löffelholz and Trendelenburg 2008; North and Hughes 2013).

**Fig. 8:**
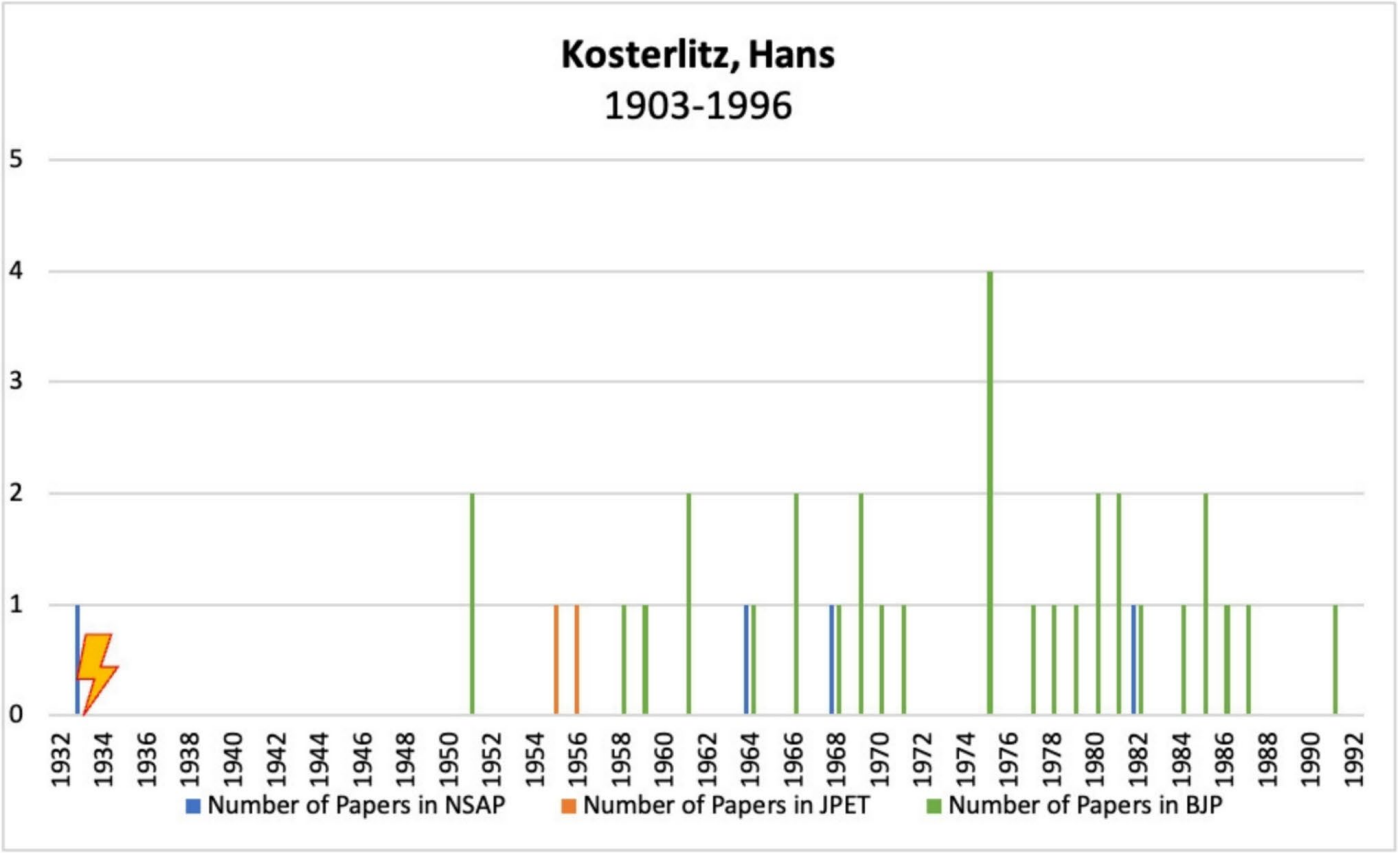
Number of publications by Hans Walter Kosterlitz in *Naunyn–Schmiedeberg’s Archives of Pharmacology*, JPET, and BJP; the lightning bolt symbolizes emigration

Figure 8 shows the publications of Hans Walter Kosterlitz in *Naunyn–Schmiedeberg’s Archives of Pharmacology*, JPET, and BJP. He published only one paper in the German journal *Naunyn–Schmiedeberg’s Archives of Pharmacology* before emigrating to Great Britain in 1933. This was followed by a hiatus of several years during which he did not publish in any of the journals analyzed here. From 1951 onwards, however, several papers appeared, two in the American *Journal of Pharmacology and Experimental Therapeutics* and three in the German journal *Naunyn–Schmiedeberg’s Archives of Pharmacology*. However, the majority of his publications appeared in the *British Journal of Pharmacology*.

Hans Walter Kosterlitz emigrated early in his scientific career and, despite the interruption, was able to achieve excellent positions and build a remarkable career in his new home.

#### Otto Krayer

Otto Krayer was born on 22 October 1899 in Köndringen, north of Freiburg im Breisgau. During the First World War, from 1917 to 1918, he served in the infantry and was wounded (Jaenicke 2004; Löffelholz and Trendelenburg 2008, Rubin 2014b).

From 1919 to 1925, he studied medicine in Freiburg, Munich, and Berlin. From 1925 to 1927, he researched under Paul Trendelenburg at the Institute of Pharmacology in Freiburg, where he obtained his doctorate in 1926.

In 1927, he moved with Paul Trendelenburg to the Institute of Pharmacology in Berlin, where he qualified as a professor in 1929. He was appointed associate professor in 1932, having already taken over the management of the Institute on an interim basis from 1930 to 1932 during Trendelenburg’s serious illness with tuberculosis and after his death (Jaenicke 2004, Löffelholz and Trendelenburg 2008, Rubin 2014, Starke 2013). During this period, the renowned scientists Marthe Louise Vogt (65 in Table S1) and Edith Bülbring (6 in Table S1) also worked at the Institute (Jaenicke 2004; Löffelholz and Trendelenburg 2008).

In the spring of 1933, Krayer received an offer of a professorship in Düsseldorf, which he declined on moral grounds. The previous holder of the chair, Philipp Ellinger, had been dismissed because of his Jewish ancestry after the “Law for the Restoration of the Professional Civil Service” came into force, and Krayer was to replace him. Krayer refused because he claimed that the dismissal of Jewish scientists was morally unacceptable (Deichmann 2002, Goldstein 1987, Rubin 2014, Starke 2013). As a result, Krayer was also initially dismissed, whereupon he emigrated to England—although his dismissal was already overturned in September 1933 (Rubin 2014). With the support of the Rockefeller Foundation, Krayer was offered a position in the Department of Pharmacology at University College in London (Goldstein 1987; Jaenicke 2004; Löffelholz and Trendelenburg 2008, Rubin 2014b).

In 1934, he left England to head the pharmacology department at the American University in Beirut, Lebanon, a position he held until 1937 (Goldstein 1987; Löffelholz and Trendelenburg 2008, Rubin 2014).

In 1937, Krayer moved to Boston, Massachusetts, where he first became Associate Professor in the Department of Pharmacology at the prestigious Harvard Medical School. In 1939, he became head of the department, and in 1951, he was appointed Professor of Pharmacology (Goldstein 1987; Löffelholz and Trendelenburg 2008, Rubin 2014).

Krayer held this position until he retired in 1966. Shortly after he retired, the institute he headed was ranked first among medical schools in the USA in an evaluation of teaching by the American Council on Education (Starke 2013).

Krayer was President of the *American Society for Pharmacology and Experimental Therapeutics* (ASPET) from 1957 to 1958 and, after his retirement, held visiting professorships at the Technical University of Munich and the University of Arizona in Tucson until shortly before his death (Jaenicke 2004, Rubin 2014).

Otto Krayer died on 18 March 1982 in Tucson, Arizona (Goldstein 1987, Jaenicke 2004, Löffelholz and Trendelenburg 2008, Rubin 2014, Starke 2013).

Krayer’s main area of research was the pharmacology of the heart and circulation (Goldstein 1987; Starke 2013).

He received many honors during his career, including honorary doctorates from the universities of Freiburg im Breisgau, Göttingen, Munich, and Harvard University. In 1964, he was awarded the Schmiedeberg Medal (Goldstein 1987; Löffelholz and Trendelenburg 2008).

**Fig. 9:**
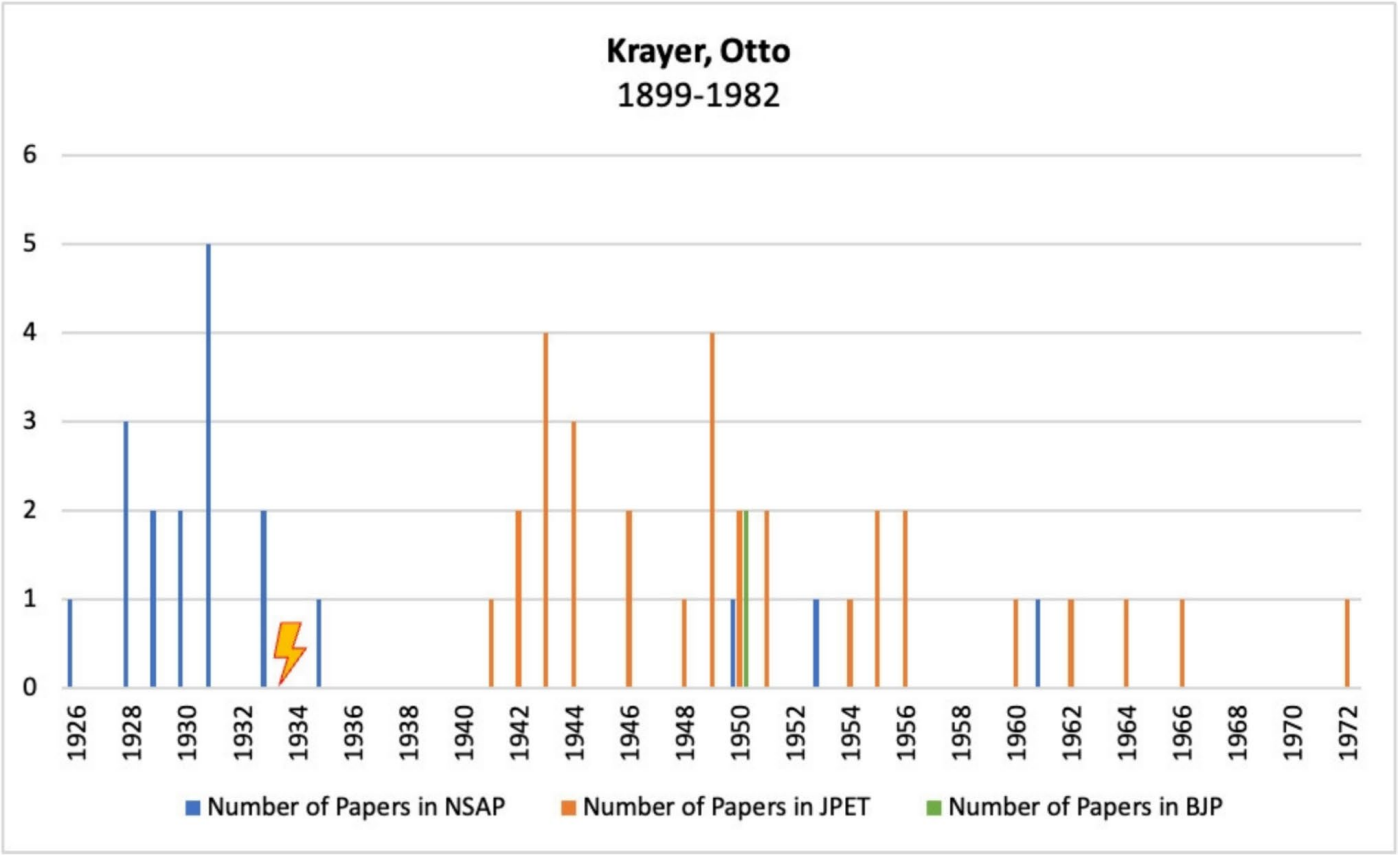
Number of publications by Otto Krayer in *Naunyn–Schmiedeberg’s Archives of Pharmacology*, JPET, and BJP; the lightning bolt symbolizes emigration

Figure 9 shows Otto Krayer’s publications in *Naunyn–Schmiedeberg’s Archives of Pharmacology*, JPET, and BJP. Before emigrating around 1933, he published numerous papers in the German journal *Naunyn–Schmiedeberg’s Archives of Pharmacology*. After he emigrated, his publication activity in this journal stagnated for 15 years, with the exception of a single publication. Instead, from 1941 onwards, many of his papers appeared in the American *Journal of Pharmacology and Experimental Therapeutics*, as Krayer had been living in the USA since 1937 (Goldstein 1987; Jaenicke 2004; Löffelholz and Trendelenburg 2008, Rubin 2014). In 1950, two of his papers were also published in *the British Journal of Pharmacology*. It is striking that, unlike many of the emigrated pharmacologists discussed here, Krayer published three more papers in the German journal *Naunyn–Schmiedeberg’s Archives of Pharmacology* after the war. His biography shows that, despite his experiences, Krayer did not turn his back on German science. From 1948 to 1953, he led the “*Medical Mission to Germany*” on behalf of the *Unitarian Service Committee*. This was the first post-war meeting of medical professors from Germany and the USA, with the aim of helping to rebuild medical education and research (Goldstein 1987, Rubin 2014, Starke 2013). In addition, as mentioned above, he became a visiting professor in Munich during the summer months for several years (Jaenicke 2004; Löffelholz and Trendelenburg 2008, Rubin 2014).

Although Krayer emigrated at an advanced stage in his career (he had already been a professor before emigrating), he was able to continue his outstanding academic career abroad and was again awarded professorships. This extraordinary career path distinguishes him from most of the pharmacologists analyzed here and underlines his importance as an outstanding scientist and teacher.

#### Otto Loewi

Otto Loewi was born in Frankfurt am Main on 3 June 1873 and studied medicine and chemistry in Munich and Strasbourg from 1891 to 1896. In 1896, under the influence of Oswald Schmiedeberg, he obtained his doctorate with a pharmacological thesis (Löffelholz and Trendelenburg 2008; Shaw and Shaw 2016; Todman 2009).

He worked as an assistant in the Department of Internal Medicine at the Municipal Hospital in Frankfurt until 1898, when he followed his desire for a scientific career and moved to the Pharmacological Institute in Marburg. There he worked under the direction of Hans Horst Meyer (44 in Table S1) and habilitated in 1900. Between 1902 and 1903, he also spent a year in the Department of Physiology at University College, London, where he met Sir Henry Hallett Dale, who was to become a lifelong friend (Dale 1962; Loewi 1960; Shaw and Shaw 2016; Todman 2009).

On his return to Marburg, Loewi took over the interim directorship of the Institute of Pharmacology for a year before moving to the Institute of Pharmacology in Vienna in 1905 together with Hans Horst Meyer, where he worked as an associate professor until 1909 (Koelle 1986; Loewi 1960; Löffelholz and Trendelenburg 2008).

In 1909, he was appointed to the chair of pharmacology at the Institute of Pharmacology in Graz. In 1936, Loewi was awarded the Nobel Prize in Physiology and Medicine together with Sir Henry Hallett Dale for the discovery of the chemical transmission of nerve impulses (Dale 1962; Koelle 1986; Loewi 1921; Todman 2009).

Following the annexation of Austria by Nazi Germany in 1938, Loewi was dismissed from his post because of his Jewish ancestry and was initially placed in “protective custody” for almost 2 months before being able to emigrate by transferring his share of the Nobel Prize to the Nazi regime. During this period of imprisonment, Loewi was particularly concerned about the publication of his latest research showing that acetylcholine does not occur in afferent nerve fibers. He was finally allowed to record his findings on a postcard, which he sent to the journal *Die Naturwissenschaften* (Loewi 1960).

In 1938, at the age of 65, Loewi emigrated to England, where he worked in the Department of Pharmacology at the Nuffield Institute in Oxford until 1940. In 1940, he moved to the USA and took up a position as Research Professor at the College of Medicine at New York University, where he continued to conduct research until his retirement in 1955 (Loewi 1960; Todman 2009).

Otto Loewi died in New York on 25 December 1961 (Dale 1962; Koelle 1986; Löffelholz and Trendelenburg 2008; Todman 2009).

Loewi’s main field of research was the pharmacology of the adrenergic and cholinergic systems (Dale 1962; Todman 2009).

In addition to the Nobel Prize in 1936, Loewi received the *Schmiedeberg-Plakette* in 1957. He was an honorary member of the *American Society for Pharmacology and Experimental Therapeutics* (ASPET) and the *German Pharmacological Society* (DPhG), was elected a “Foreign Member of the Royal Society” in 1954, and received honorary doctorates from the universities of Graz, Yale, New York, and Frankfurt (Loewi 1960; Löffelholz and Trendelenburg 2008; Shaw and Shaw 2016).

**Fig. 10:**
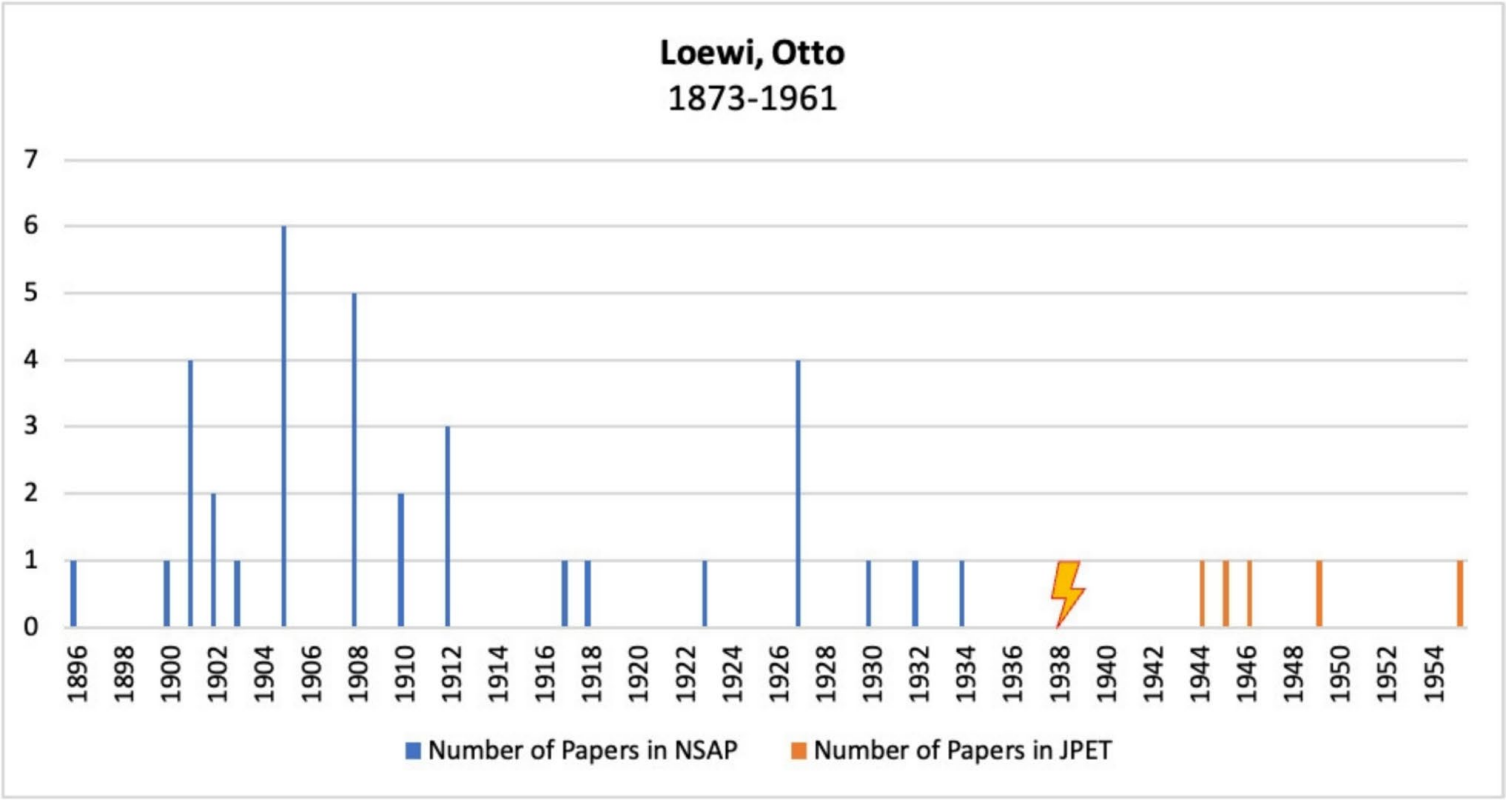
Number of publications by Otto Loewi in *Naunyn–Schmiedeberg’s Archives of Pharmacology*, JPET, and BJP; the lightning bolt symbolizes emigration

Figure 10 shows Otto Loewi’s publications in *Naunyn–Schmiedeberg’s Archives of Pharmacology*, JPET, and BJP. Before emigrating, Loewi published a considerable number of articles in the German journal *Naunyn–Schmiedeberg’s Archives of Pharmacology*. However, after emigrating, no further publications appeared in this journal. Instead, after emigrating to the USA, he published five articles in the American *Journal of Pharmacology and Experimental Therapeutics*. No papers by him appeared in the *British Journal of Pharmacology*.

Loewi had already won the Nobel Prize before he was dismissed from his post as professor at the Institute of Pharmacology in Graz. He was able to obtain a new professorship after emigrating. This makes him—along with Otto Krayer—one of the few expelled pharmacologists analyzed here who were able to obtain an equivalent academic position abroad after being dismissed from a professorship in Germany.

Loewi himself described it as “good luck” that he got a professorship in New York and emphasized that he was “deeply grateful to the fate that transported […] [him] to this country” (Loewi 1960 25).

#### Hans Molitor

Hans Molitor was born on 10 August 1895 in Maffersdorf, Bohemia, and studied medicine and pharmacy at the University of Vienna. From 1922, he worked at the Institute of Pharmacology in Vienna, first under Hans Horst Meyer (44 in Table S1) and later under Ernst Peter Pick (50 in Table S1). He qualified as a professor in 1927 and was appointed associate professor in 1931 (Löffelholz and Trendelenburg 2008, The New York Times 1970).

Hans Molitor emigrated to the USA in 1932 at the age of 37. In 1933, he was recruited by Merck & Co. to head the newly established *Merck Institute of Therapeutic* *Research* in Rahway, New Jersey. He played a key role in the establishment of the Institute and had a decisive influence on the development of pharmaceutical research at Merck & Co. Under his leadership, key research priorities were defined that strengthened the company in the long term. Molitor held this position until his retirement in 1960 (Godley et al. 2019; Gortler 2000; Löffelholz and Trendelenburg 2008).

After his retirement, he served for 4 years as President of the *Society for the Promotion of International Relations* (The New York Times 1970).

Hans Molitor died in Bricktown on 5 August 1970 (Löffelholz and Trendelenburg 2008, The New York Times 1970).

Molitor was an honorary member of the German Pharmacological Society (DPhG) (Löffelholz and Trendelenburg 2008).

In 1953, he was the first scientist to receive an award from the *American Society of European Chemists* in recognition of his special commitment to international scientific relations (The New York Times 1970).

Because of his Jewish origins, his habilitation was revoked in Germany in 1938 (Löffelholz and Trendelenburg 2008).

**Fig. 11:**
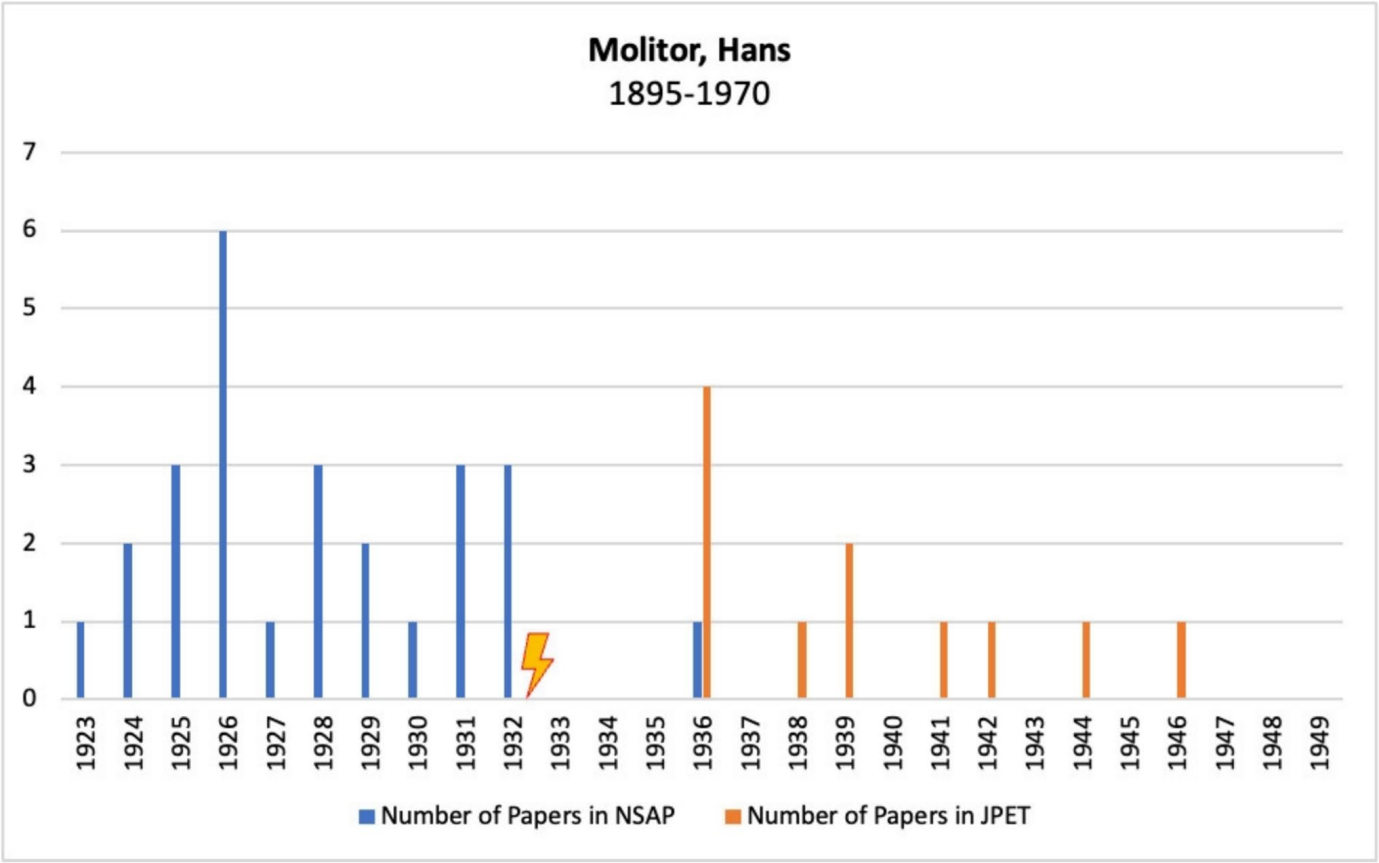
Number of publications by Hans Molitor in *Naunyn–Schmiedeberg’s Archives of Pharmacology*, JPET, and BJP; the lightning bolt symbolizes emigration

Figure 11 shows Hans Molitor’s publications in *Naunyn–Schmiedeberg’s Archives of Pharmacology*, JPET, and BJP.

Before emigrating, he published regularly in the German journal *Naunyn–Schmiedeberg’s Archives of Pharmacology*. After emigrating to the USA, his papers appeared mainly in the *Journal of Pharmacology and Experimental Therapeutics*, with one exception published in the German journal.

Before emigrating, Molitor worked as an associate professor in academia, and after arriving in the USA, he became the director of a large private research institute. Unlike the other pharmacologists analyzed here, he did not emigrate because of discrimination or expulsion, but to take up a new professional challenge. Despite his absence, his habilitation at the University of Vienna was revoked in 1938 after Austria’s annexation by Germany (Löffelholz and Trendelenburg 2008). However, this did not affect his career in the USA.

#### Ernst Peter Pick

Ernst Peter Pick was born in Jaromer, Bohemia, on 18 May 1872. He studied medicine at the University of Prague and graduated in 1896.

From 1896 to 1899, he worked at the Institute of Physiological Chemistry at the University of Strasbourg. He then worked at the State Serum Institute in Vienna until 1911, where he was involved in the production of vaccines (Jenss 2022; Löffelholz and Trendelenburg 2008).

In 1911, he moved to the Pharmacological Institute in Vienna, where he conducted research under the direction of Hans Horst Meyer (44 in Table S1) and was appointed associate professor in 1912 (Jenss 2022; Löffelholz and Trendelenburg 2008).

In 1919, he qualified as a professor of experimental pharmacology, and in 1924, he was appointed full professor and took over the directorship of the institute as Hans Horst Meyer’s successor. In addition to his work as director of the institute, Pick was Dean and Vice-Dean of the Faculty of Medicine from 1932 to 1937. From 1914 to 1938, he also headed the Research Institute of the National Health Authority in Vienna (Jenss 2022; Löffelholz and Trendelenburg 2008).

With the annexation of Austria by Nazi Germany in 1938, Pick was forced to retire and—like his predecessor Hans Horst Meyer—had to move into a barrack (Löffelholz and Trendelenburg 2008; Sienell and Feldmann 2011).

That same year, at the age of 66, Pick emigrated to the USA. From 1939, he worked as a clinical professor of pharmacology at Columbia University in New York and at Mount Sinai Hospital. He also worked as a consultant at the Merck Institute of Therapeutic Research in Rahway, New Jersey, which was then headed by Hans Molitor (45 in Table S1) (Jenss 2022; Löffelholz and Trendelenburg 2008).

During his emigration and search for a new job, Pick was assisted by Klaus Unna (64 in Table S1), who had already emigrated to the USA in 1937 (Jenss 2022; Löffelholz and Trendelenburg 2008).

Pick retired in 1946 and died in New York on 15 January 1960 (Jenss 2022; Löffelholz and Trendelenburg 2008).

Ernst Peter Pick received numerous honors, including an honorary doctorate from the University of Vienna in 1952, the Schmiedeberg Medal in 1957, and the Silver Medal of Merit of the Republic of Austria in the same year (Löffelholz and Trendelenburg 2008).

**Fig. 12:**
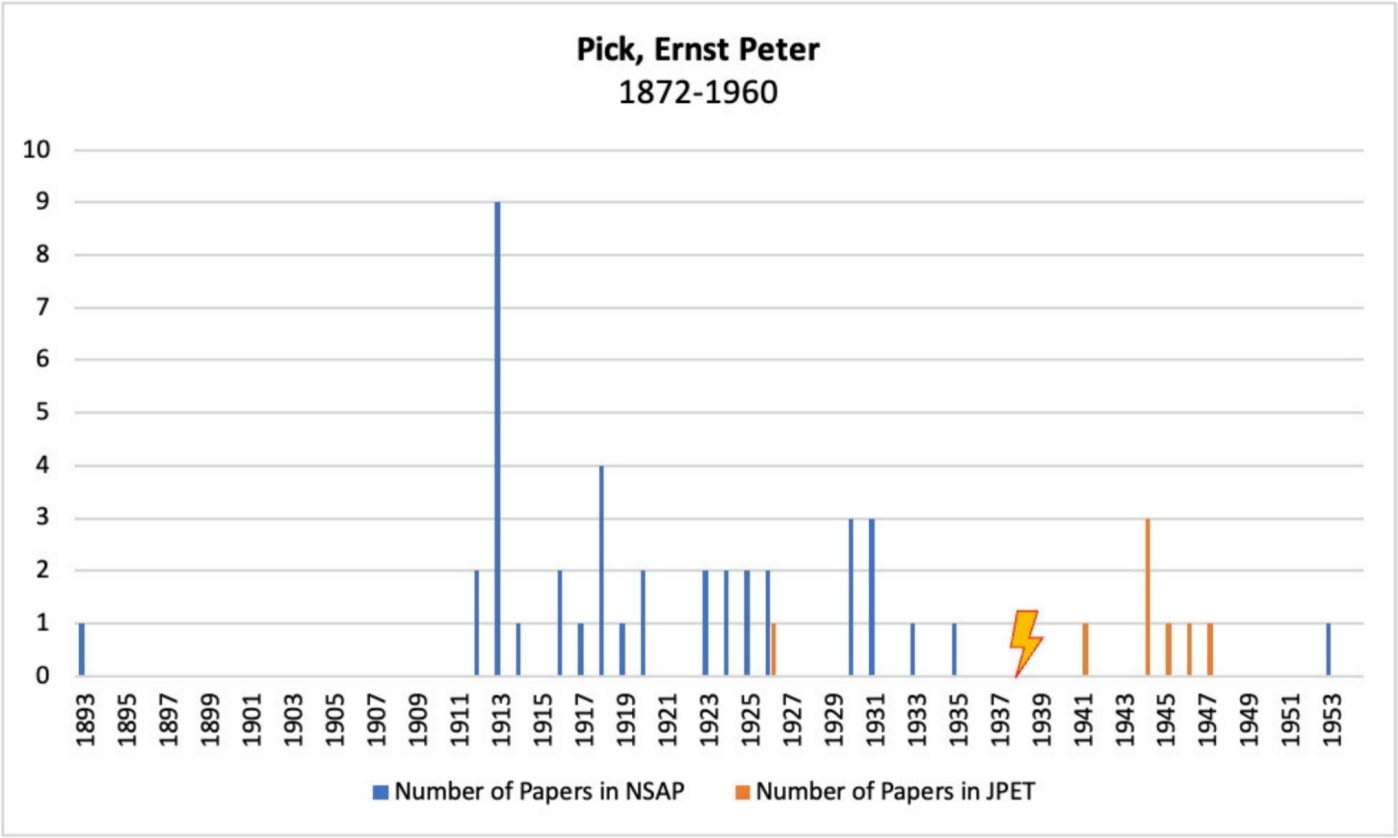
Number of publications by Ernst Peter Pick in *Naunyn–Schmiedeberg’s Archives of Pharmacology*, JPET, and BJP; the lightning bolt symbolizes emigration

Figure 12 shows Ernst Peter Pick’s publications in *Naunyn–Schmiedeberg’s Archives of Pharmacology*, JPET, and BJP. Before emigrating to the USA in 1938, Pick published numerous articles in the German journal *Naunyn–Schmiedeberg’s Archives of Pharmacology*. After emigrating, he also published in the American *Journal of Pharmacology and Experimental Therapeutics*, but to a much lesser extent than in the German journal. Furthermore, only one article appeared in *Naunyn–Schmiedeberg’s Archives of Pharmacology* after his emigration.

Despite his expulsion from Austria, Pick was able to obtain an equivalent academic position in the USA.

#### Otto Riesser

Otto Riesser was born in Frankfurt am Main on 7 September 1882. From 1901, he studied chemistry at the Universities of Berlin and Heidelberg, gaining a doctorate in chemistry from Heidelberg in 1906. From 1903, he also studied medicine, graduating in 1908 in Heidelberg and Berlin (Be̜benek-Gerlich 2009, Löffelholz and Trendelenburg 2008; Taubmann 1950).

Riesser then worked at the Pharmacological Institute of the University of Königsberg from 1908 to 1914. During this time, he obtained his doctorate in medicine in 1911 and habilitated in medicinal chemistry in 1913 (Be̜benek-Gerlich 2009, Löffelholz and Trendelenburg 2008; Taubmann 1950).

At the outbreak of the First World War in 1914, Riesser took on various military medical duties. From 1915 to 1919, he also worked at the Pharmacological Institute in Frankfurt under the direction of Alexander Ellinger, with whom he had already done research in Königsberg. He habilitated in pharmacology in Frankfurt in 1916 and was appointed extraordinary professor in 1918 (Be̜benek-Gerlich 2009, Löffelholz and Trendelenburg 2008; Taubmann 1950).

From 1919, Riesser worked for 2 years at the Institute of Vegetative Physiology in Frankfurt, where he habilitated in physiology (Be̜benek-Gerlich 2009, Löffelholz and Trendelenburg 2008; Taubmann 1950).

In 1921, he accepted a chair of pharmacology at the University of Greifswald. In 1928, he succeeded Otto Loewi as chair of pharmacology at the University of Breslau (Be̜benek-Gerlich 2009, Löffelholz and Trendelenburg 2008; Taubmann 1950).

In the course of the National Socialist takeover, Riesser was dismissed as director of the Institute in 1934 following the “Law for the Restoration of the Professional Civil Service”. He was initially allowed to continue his research at the Institute for another year while he awaited the official implementation of his forced transfer (Be̜benek-Gerlich 2009, Löffelholz and Trendelenburg 2008; Starke 1998; Taubmann 1950).

In 1935, he was assigned a laboratory in the Georg-Speyer-Haus in Frankfurt, which was officially approved by the Ministry of Education. However, only 3 days after his move to Frankfurt, the Nuremberg Laws came into force, further restricting his rights as a Jew (Be̜benek-Gerlich 2009, Essner 2002; Friedländer 2007). Riesser then had to return to Breslau, where he was finally relieved of all official duties (Be̜benek-Gerlich 2009).

In the same year, Riesser received an offer from the *Swiss Research Institute for High Mountain Climate and Medicine* in Davos to lead the reorganization of the physiological-chemical department there. However, he had to give up this position only a year later when his request to extend his stay abroad was rejected by the Ministry in Berlin (Be̜benek-Gerlich 2009).

Riesser returned to Germany in 1937 and found a position in the laboratory of his cousin Ferdinand Blum (Be̜benek-Gerlich 2009, Taubmann 1950).

During the November pogroms of 1938, Riesser was arrested and officially ordered to leave Germany (Be̜benek-Gerlich 2009, Hermann 2008).

In 1939, at the age of 57, he emigrated to the Netherlands, where he was able to continue his research at the Pharmacotherapeutical Institute in Amsterdam under the direction of Ernst Laqueur (34 in Table S1) (Be̜benek-Gerlich 2009, Löffelholz and Trendelenburg 2008; Starke 1998; Taubmann 1950).

After the German invasion of the Netherlands in 1941, Riesser, like Laqueur, was again dismissed from his post because of his Jewish origins. He then set up a small private laboratory in Naarden-Bussum, but was hardly able to work and research effectively due to massive financial difficulties (Be̜benek-Gerlich 2009, Löffelholz and Trendelenburg 2008).

After the end of the Second World War, Riesser returned to Germany in 1945 (Be̜benek-Gerlich 2009, Löffelholz and Trendelenburg 2008; Taubmann 1950). Since academic work was hardly possible in war-ravaged Germany, he initially took up a position as special adviser for culture and education at the *Großhessisches Staatsministerium* (Ministry of State of Greater Hesse) in 1945 (Be̜benek-Gerlich 2009, Löffelholz and Trendelenburg 2008, Starke 1998, Taubmann 1950).

In 1946, he was able to resume his academic career when he was offered a lectureship in the border area between physiology and pharmacology at the University of Frankfurt (Be̜benek-Gerlich 2009, Taubmann 1950).

In 1947, Riesser was elected chairman of the *German Pharmacological Society* (Be̜benek-Gerlich 2009, Löffelholz and Trendelenburg 2008).

At the beginning of 1949, he also became provisional director of the Institute of Pharmacology at the University of Frankfurt (Be̜benek-Gerlich 2009, Löffelholz and Trendelenburg 2008). In the same year, on 1 December 1949, Otto Riesser died during surgery for a duodenal ulcer (Be̜benek-Gerlich 2009, Taubmann 1950).

Riesser’s main field of research was the physiology of muscles (Be̜benek-Gerlich 2009, Taubmann 1950).

**Fig. 13:**
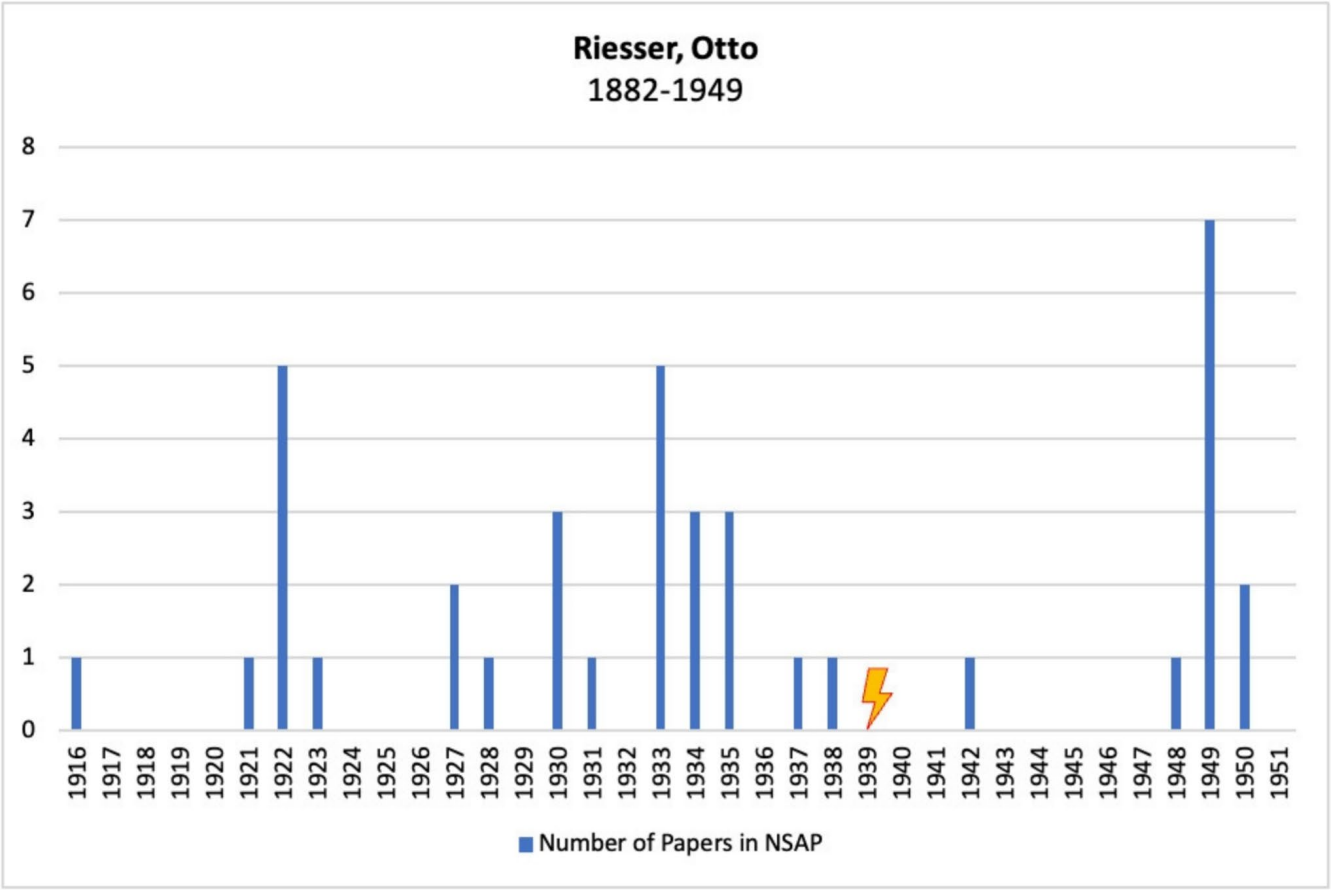
Number of publications by Otto Riesser in *Naunyn–Schmiedeberg’s Archives of Pharmacology*, JPET, and BJP; the lightning bolt symbolizes emigration

Figure 13 shows Otto Riesser’s publications in *Naunyn–Schmiedeberg’s Archives of Pharmacology*, JPET, and BJP. Of the journals analyzed here, Riesser published exclusively in *Naunyn–Schmiedeberg’s Archives of Pharmacology.*

The graph shows that Riesser published numerous articles in this German journal until his forced emigration in 1939—even after the Nazi seizure of power in 1933. It became increasingly difficult for Jewish scientists to publish in German journals. Especially after the Nuremberg Laws were passed in 1935, Jewish scientists were systematically excluded from academic life, which makes Riesser’s continued publication activity in the *Naunyn–Schmiedeberg’s Archives of Pharmacology* (Beddies et al. 2014; Essner 2002; Friedländer 2007; Medawar and Pyke 2001) all the more remarkable.

It is also noteworthy that Riesser published another article in this journal in 1942 during his time at the Pharmacotherapeutic Institute in Amsterdam. Given the political situation in Germany and the systematic suppression of Jewish scientists from the scientific community, this late publication is an absolute exception (Be̜benek-Gerlich 2009, Mispagel and Seifert 2024; Taubmann 1950).

Riesser was one of the few persecuted pharmacologists to return to Germany after emigrating (Be̜benek-Gerlich 2009, Mispagel and Seifert 2024; Taubmann 1950). Despite the massive discrimination and persecution he experienced as a scientist of Jewish descent, he maintained his deep attachment to Germany and was particularly conciliatory towards his former Nazi colleagues (Be̜benek-Gerlich 2009).

This attitude is also reflected in Fig. 11: Riesser is one of the few pharmacologists analyzed here who continued to publish in the *Naunyn–Schmiedeberg’s Archives of Pharmacology* after emigrating. He also managed to obtain a comparable academic position after his emigration.

#### Klaus Robert Walter Unna

Klaus Robert Walter Unna was born on 30 July 1908 in Hamburg. He received his doctorate in medicine from the University of Freiburg in 1930 and then completed a 2-year internship in Cologne, Berlin, and Hamburg (Löffelholz and Trendelenburg 2008).

In 1932, he took up a post at the Balneological Institute in Bad Oeynhausen. After the Nazi seizure of power in 1933, he left Germany and went to Austria, as he was classified as “non-Aryan” (Büttner 1989; Löffelholz and Trendelenburg 2008).

From 1933 to 1937, he worked at the Pharmacological Institute of the University of Vienna under the direction of Ernst Peter Pick (50 in Table S1). In 1937, Unna emigrated to the USA and worked until 1944 at the Merck Institute of Therapeutic Research in Rahway, New Jersey, headed by Hans Molitor (45 in Table S1) (Löffelholz and Trendelenburg 2008).

In 1944, he joined the Department of Pharmacology at the University of Pennsylvania in Philadelphia as an instructor. A year later, he accepted a position in the Department of Pharmacology at the University of Illinois College of Medicine in Chicago, first as an assistant professor and later as an associate professor. He was promoted to full professor in 1950 and headed the department from 1955 until his retirement in 1976 (Löffelholz and Trendelenburg 2008).

In addition to his academic activities, Unna was secretary of the International Brain Research Organisation (IBRO) from 1965 to 1969. In 1970, he was made an honorary member of the German Pharmacological Society (Löffelholz and Trendelenburg 2008).

Klaus Robert Walter Unna died on 26 June 1987 in Santa Fe, New Mexico (Löffelholz and Trendelenburg 2008).

**Fig. 14:**
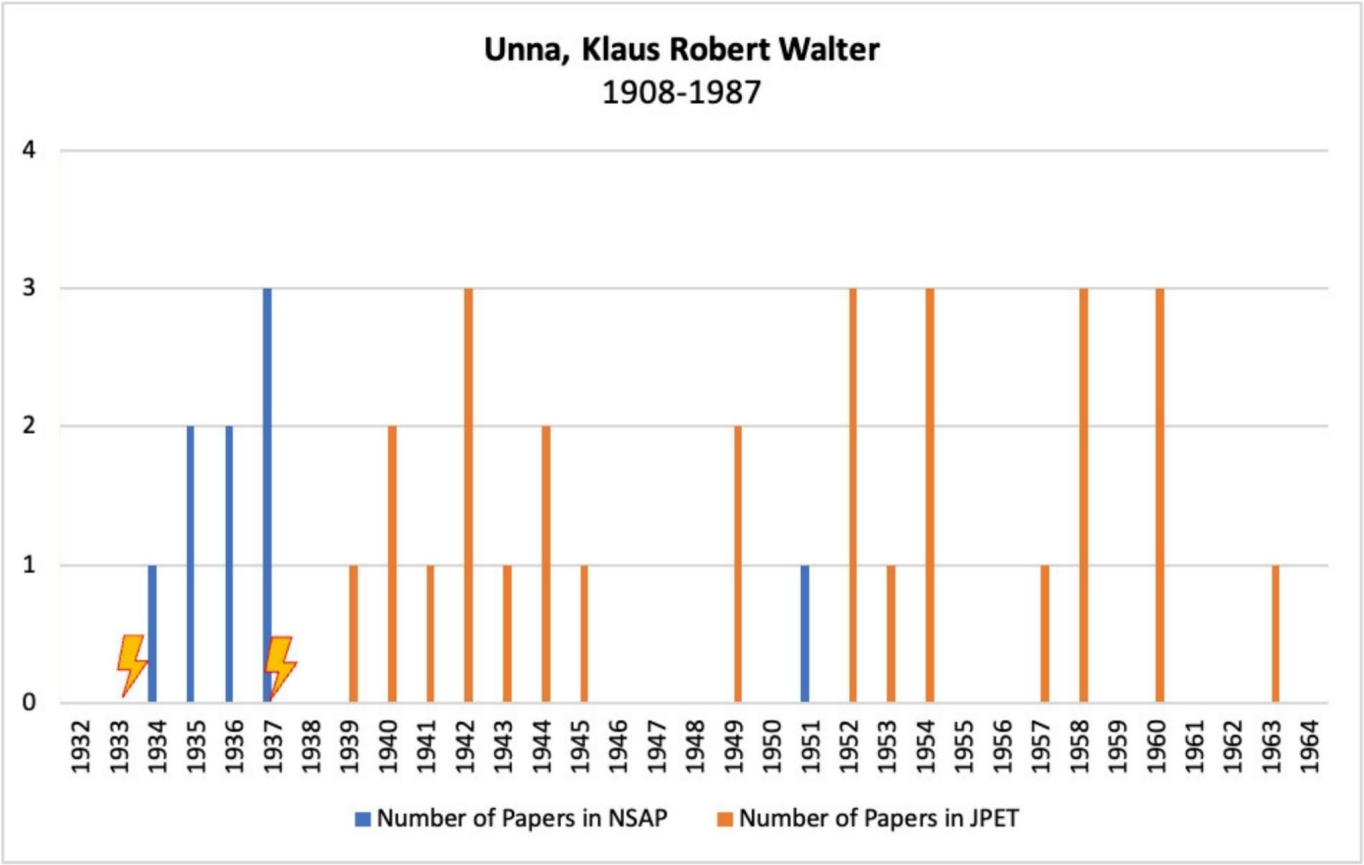
Number of publications by Klaus Robert Walter Unna in *Naunyn–Schmiedeberg’s Archives of Pharmacology*, JPET, and BJP; the lightning bolt symbolizes emigration

Figure 14 shows Klaus Robert Walter Unna’s publications in *Naunyn–Schmiedeberg’s Archives of Pharmacology*, JPET, and BJP.

Unna emigrated to Austria early in his scientific career, but continued to publish in the German journal *Naunyn–Schmiedeberg’s Archives of Pharmacology*. He published a total of eight articles in this journal during this period. Given the increasing discrimination against Jewish scientists, only a few of the persecuted pharmacologists analyzed here were able to continue publishing in *Naunyn–Schmiedeberg’s Archives of Pharmacology* at this time.

In 1937, shortly before the “Anschluss” of Austria to Germany, Unna emigrated to the USA (Löffelholz and Trendelenburg 2008). From then on, he published mainly in the *Journal of Pharmacology and Experimental Therapeutics*, where he wrote a total of 28 articles. Only in 1951 did a single article by him appear in *Naunyn–Schmiedeberg’s Archives of Pharmacology*. However, it was labelled as having been written in 1937, but was not published until 1951 due to “external reasons” (Unna 1951). This is a striking illustration of the restrictions to which Jewish scientists were subjected during the Nazi era: scientific papers could often no longer be published or, as in this example, were published only after many years of delay.

At the time of his emigration, Unna was still at the beginning of his scientific career. With the support of Hans Molitor (45 in Table S1), who had also fled Germany, he was able to quickly establish himself in the US and eventually held the chair of the Department of Pharmacology at the University of Illinois College of Medicine for many years (Löffelholz and Trendelenburg 2008).

#### Marthe Louise Vogt

Marthe Louise Vogt was born on 8 September 1903, the daughter of Oskar and Cécile Vogt, two of the leading brain researchers of their time (Cuthbert 2005; Klatzo 2002; Löffelholz and Trendelenburg 2008, Rubin 2017).

She studied medicine and chemistry in Berlin from 1922 to 1929, obtaining a doctorate in medicine in 1928 and a doctorate in chemistry in 1929. In 1929, she took up a post at the Pharmacological Institute in Berlin under the direction of Paul Trendelenburg. From 1931, she worked as a research assistant and later as head of the chemical department at the Kaiser Wilhelm Institute for Brain Research in Berlin, where her father was director (Cuthbert 2005; Löffelholz and Trendelenburg 2008, Rubin 2017).

Although Vogt was not of Jewish descent, she rejected the political developments in Germany. In 1935, she emigrated to Great Britain on a Rockefeller Fellowship (Cuthbert 2005; Löffelholz and Trendelenburg 2008,  Rubin 2017).

In Great Britain, she first worked at the *National Institute of Medical Research (NIMR)* in Hampstead, London, under the direction of Sir Henry Hallett Dale. There she met Wilhelm Feldberg (11 in Table S1), who had also emigrated from Germany.

From 1936, Vogt spent the second half of her fellowship at the Department of Pharmacology in Cambridge. Thanks to the support of the head of the department, E B Verney, she received further funding which enabled her to remain in Great Britain (Cuthbert 2005; Löffelholz and Trendelenburg 2008, Rubin 2017).

After her fellowships expired in 1941, Vogt obtained a position in the Pharmacological Laboratory of the Pharmaceutical Society in London, one of the leading centers of pharmacology in Britain at the time (Cuthbert 2005; Löffelholz and Trendelenburg 2008, Rubin 2017).

In 1947, she became a lecturer in the Department of Pharmacology in Edinburgh, rising to Senior Lecturer in 1951 and Reader in 1952. In the same year, she was elected a Fellow of the Royal Society, an honor that had previously been bestowed on only eight other women. With a short interruption for a sabbatical at Columbia University Medical School in New York, she remained in Edinburgh until 1960 (Cuthbert 2005; Löffelholz and Trendelenburg 2008, Rubin 2017).

In 1960, Vogt became head of the pharmacology unit of the Animal Research Council Institute of Animal Physiology at Babraham, near Cambridge. Although she officially retired in 1968, she remained active there, publishing scientific papers until the 1980 s (Cuthbert 2005; Löffelholz and Trendelenburg 2008, Rubin 2017).

When her eyesight began to fail and she was threatened with blindness, Marthe Vogt moved to San Diego, California in 1990 to be near her sister. She died there on 9 September 2003, the day after her 100 th birthday (Cuthbert 2005; Löffelholz and Trendelenburg 2008, Rubin 2017).

Marthe Vogt’s main areas of research were neuropharmacology, in particular the role of acetylcholine and catecholamines in signal transmission, and the function of the adrenal cortex and its influence on stress responses. She has also worked on circulatory regulation and the pathophysiology of hypertension. (Cuthbert 2005, Rubin 2017).

In addition to being elected a Fellow of the Royal Society, she received an honorary doctorate from the University of Cambridge in 1975 and the *Schmiedeberg-Plakette* in 1981 (Cuthbert 2005; Löffelholz and Trendelenburg 2008, Rubin 2017).

**Fig. 15:**
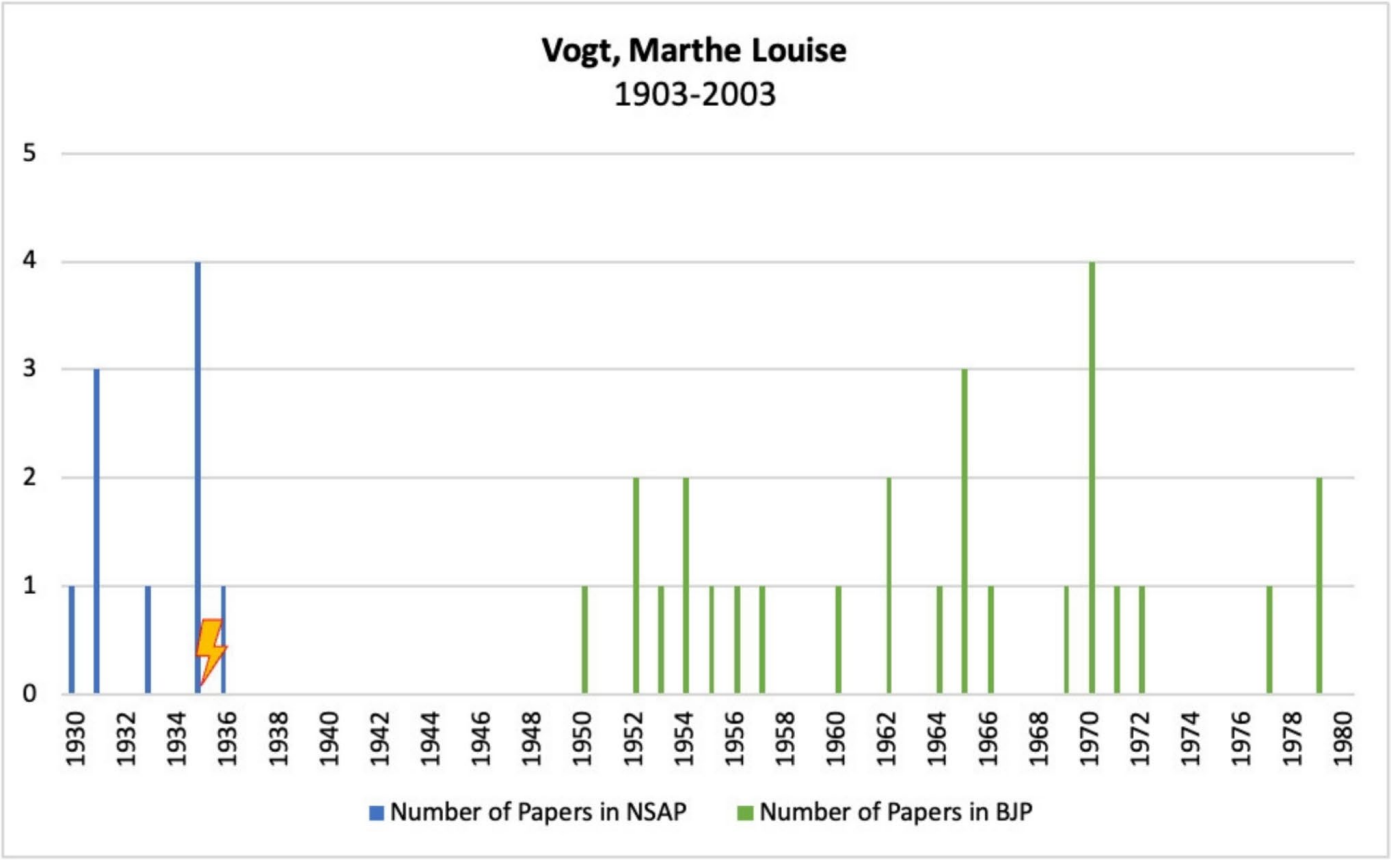
Number of publications by Marthe Louise Vogt in *Naunyn–Schmiedeberg’s Archives of Pharmacology*, JPET, and BJP; the lightning bolt symbolizes emigration

Figure 15 shows Marthe Louise Vogt’s publications in *Naunyn–Schmiedeberg’s Archives of Pharmacology*, JPET, and BJP.

Before emigrating to Great Britain, Vogt published nine articles in *Naunyn–Schmiedeberg’s Archives of Pharmacology*. After her emigration, except for a single article in 1936, no further work appeared there. It was only after a 14-year hiatus that she published her first paper in the *British Journal of Pharmacology* in 1950.

Over the course of her career, a total of 27 articles followed in the *British Journal of Pharmacology*, more than a third of which appeared after her official retirement.

Vogt used her fellowship to establish herself as a scientist in the UK. Thanks to a strong academic network, excellent research results and targeted funding opportunities, she was able to build a successful long-term career there.

In summary, Vogt’s emigration paved the way for a significant international career, which would probably not have been possible in Germany due to her political stance under the Nazi regime.

## Conclusion

An analysis of the professional development of persecuted pharmacologists before and after emigration reveals a complex picture: While some scientists were able to establish and continue significant academic careers despite forced migration, others suffered significant setbacks.

In particular, young scientists in the early stages of their careers were often able to establish themselves well in their host countries. High-ranking academics with professorships or heads of institutes, on the other hand, often found it difficult to find a comparable position after emigration.

The detailed biographies presented here show that factors such as academic reputation, personal commitment, and existing networks in exile had a decisive influence on the chances of professional reintegration.

It also illustrates the long-term effects of Nazi persecution on the academic landscape, both in the countries of origin and in the countries of refuge.

## Limitations

This study focuses primarily on the biographies of particularly successful scientists, most of whom were able to pursue their careers despite adverse circumstances. It therefore does not represent a complete cross-section of all persecuted pharmacologists.

In particular, scientists who were unable to pursue an academic career after emigrating or whose careers were severely curtailed are under-represented.

Further studies are needed to provide a more holistic picture of the impact of persecution and exile on pharmacology. Future research should also include lesser known or forgotten scientists to gain a complete overview of the individual fates and structural conditions of academic migration during this period.

## Supplementary Information

Below is the link to the electronic supplementary material.

Supplementary file1 (DOCX 23 KB)

## Data Availability

All source data for this study are available upon reasonable request from the authors.
