# Supplementary material for: Biographical analysis of 32 pharmacologists persecuted under the Nazi regime: scientific careers between persecution, emigration, and new beginnings
Source: Naunyn Schmiedebergs Arch Pharmacol. 2025 Jun 9;398(12):17277–302. doi: 10.1007/s00210-025-04231-7 (PMC12678629; doi:10.1007/s00210-025-04231-7)
Supplement: Supplementary file 1 — Supplementary file1 (DOCX 23 KB) [file 210_2025_4231_MOESM1_ESM.docx]

**Biographical analysis of 32 pharmacologists persecuted under the Nazi regime: Scientific careers between persecution, emigration and new beginnings**

**Supplements**

**Mirja Mispagel^1^, Roland Seifert^1^**

^1^Institute of Pharmacology

Hannover Medical School

Carl-Neuberg-Str. 1

D-30625 Hannover, Germany

Correspondence: [seifert.roland@mh-hannover.de](mailto:seifert.roland@mh-hannover.de)

**Keywords**

National Socialism ⋅ Third Reich ⋅ *Naunyn-Schmiedeberg‘s Archives of Pharmacology* ⋅ Persecuted Pharmacologists ⋅ Publication Activity ⋅ Biographies ⋅ *British Journal of Pharmacology* ⋅ *Journal of Pharmacology and Experimental Therapeutics*

|  | **Persecuted Pharmacolgist (family name, given name)** | **Year of Birth** | **Year of Death** | **Gender** | **Degree programmes** | **Destiny** | **Emigration year** | **Emigration to** |
| --- | --- | --- | --- | --- | --- | --- | --- | --- |
| **1** | Bergmann, Felix Eliezer | 1908 | 2002 | m | Medicine, Chemistry | Emigration | 1933 | Palestine |
| **2** | Blaschko, Hermann Karl Felix | 1900 | 1993 | m | Medicine | Emigration | 1933 | GB |
| **3** | Born, Gustav Victor Rudolf | 1921 | 2018 | m | Medicine | Emigration | 1933 | GB |
| **4** | Brauer, Ralph Werner | 1921 | 2000 | m | Chemistry, Biochemistry | Emigration | 1937 | USA |
| **5** | Bueding, Ernst B. (alias Ernest B.) | 1910 | 1986 | m | Medicine | Emigration | 1933 | USA |
| **6** | Bülbring, Edith | 1903 | 1990 | f | Medicine | Emigration | 1933 | GB |
| **7** | Copley, Alfred Lewin | 1910 | 1992 | m | Medicine | Emigration | 1935 | USA |
| **8** | Dresel, Peter | 1925 | 1987 | m | Chemistry, Biology | Emigration | 1938 | USA |
| **9** | Ellinger, Friedrich P. | 1900 | 1962 | m | Medicine | Emigration | 1936 | USA |
| **10** | Ellinger, Philipp | 1887 | 1952 | m | Medicine, Chemistry | Emigration | 1933 | GB |
| **11** | Feldberg, Wilhelm Siegmund | 1900 | 1993 | m | Medicine | Emigration | 1933 | GB |
| **12** | Forst, August Wilhelm | 1890 | 1981 | m | Medicine, Chemistry | Stayed in Germany | / |  |
| **13** | Freund, Ernst | 1863 | 1946 | m | Medicine, Chemistry | Emigration | 1938 | GB |
| **14** | Freund, Hermann | 1882 | 1944 | m | Medicine, Chemistry | Murder in Concentration Camp (1944) | 1940 | Netherlands |
| **15** | Fröhlich, Alfred | 1871 | 1953 | m | Medicine | Emigration | 1939 | USA |
| **16** | Glaubach, Susi | 1893 | 1964 | f | Chemistry | Emigration | 1938 | USA |
| **17** | Grab, Werner | 1903 | 1965 | m | Medicine | Stayed in Germany | / |  |
| **18** | Griesbach, Walter Edwin | 1888 | 1968 | m | Medicine | Emigration | 1939 | New Zealand |
| **19** | Handovsky, Hans | 1888 | 1959 | m | Chemistry | Emigration | 1933 | Belgium |
| **20** | Hausmann, Walther | 1877 | 1938 | m | Medicine | Suicide (1938) | / |  |
| **21** | Havemann, Robert | 1910 | 1982 | m | Chemistry | Stayed in Germany | / |  |
| **22** | Heller, Hans Sigmund | 1905 | 1974 | m | Chemistry | Emigration | 1934 | GB |
| **23** | Hellmann, Kurt | 1922 | 2013 | m | Chemistry | Emigration | 1933 | GB |
| **24** | Henze, Carlo | 1907 | 2003 | m | Medicine | Emigration | 1938/39 | USA |
| **25** | Herxheimer, Andrew | 1925 | 2016 | m | Medicine | Emigration | 1938 | GB |
| **26** | Herxheimer,  Herbert Gotthold Joachim | 1894 | 1985 | m | Medicine | Emigration | 1938 | GB |
| **27** | Holz, Siegbert | 1911 | 1999 | m | Medicine | Emigration | 1933 | Venezuela |
| **28** | Jacoby, Martin Johann | 1872 | 1941 | m | Medicine | Emigration | 1939 | GB |
| **29** | Kapeller-Adler, Regina | 1900 | 1991 | f | Chemistry | Emigration | 1939 | GB |
| **30** | Kochmann, Martin | 1878 | 1936 | m | Medicine | Suicide in Prison (1936) | / |  |
| **31** | Kohn, Richard (alias Richards, Richard Kohn) | 1904 | 1983 | m | Medicine | Emigration | 1935 | USA |
| **32** | Kosterlitz, Hans | 1903 | 1996 | m | Medicine | Emigration | 1933 | GB |
| **33** | Krayer, Otto | 1899 | 1982 | m | Medicine | Emigration | 1933 | USA |
| **34** | Laqueur, Ernst | 1880 | 1947 | m | Medicine, Chemistry | Stayed in Germany |  |  |
| **35** | Lehr, David | 1910 | 2010 | m | Medicine | Emigration | 1938 | USA |
| **36** | Lipschitz, Werner Ludwig | 1892 | 1948 | m | Medicine, Chemistry | Emigration | 1933 | USA |
| **37** | Loewe, Siegfried Walter | 1884 | 1963 | m | Medicine | Emigration | 1933 | USA |
| **38** | Loewi, Otto | 1873 | 1961 | m | Medicine, Chemistry | Emigration | 1938/39 | USA |
| **39** | Maengwyn-Davies, Gertrude Diane | 1910 | 1985 | f | Pharmacy, Chemistry | Emigration | 1938 | USA |
| **40** | Marquardt, Peter | 1910 | 1997 | m | Medicine, Chemistry | Stayed in Germany |  |  |
| **41** | Mautner, Hans | 1886 | 1963 | m | Medicine | Emigration | 1938 | USA |
| **42** | Mautner, Henry G. | 1925 | 1995 | m | Chemistry | Emigration | 1938 | USA |
| **43** | Meier, Rolf | 1897 | 1966 | m | Medicine | Emigration | 1935 | Switzerland |
| **44** | Meyer, Hans Horst | 1853 | 1939 | m | Medicine, Chemistry | Stayed in Germany | / |  |
| **45** | Molitor, Hans | 1895 | 1970 | m | Pharmacy, Medicine | Emigration | 1932 | USA |
| **46** | Müller, Franz | 1871 | 1945 | m | Medicine, Chemistry | Emigration | 1935/37 | Brazil |
| **47** | Noether, Paul | 1888 | 1933 | m | Chemistry | Suicide (1933) | / |  |
| **48** | Oppenheimer, Ernst | 1888 | 1962 | m | Medicine | Emigration | 1936 | USA |
| **49** | Peters, Georg (alias Peters, Georges) | 1920 | 2006 | m | Medicine, Biochemistry | Emigration | 1937/38 | Turkey |
| **50** | Pick, Ernst Peter | 1872 | 1960 | m | Medicine | Emigration | 1938 | USA |
| **51** | Pietrkowski, Georg (alias Peters, George) | 1874 | 1964 | m | Medicine | Emigration | 1933 | USA |
| **52** | Pollak, Leo | 1878 | 1946 | m | Medicine | Emigration | 1939 | GB |
| **53** | Pulewka, Paul | 1896 | 1989 | m | Medicine | Emigration | 1935 | Turkey |
| **54** | Riesser, Otto | 1882 | 1949 | m | Medicine, Chemistry | Emigration | 1939 | Netherlands |
| **55** | Rosenberg, Walter (alias Rudolf Vrba) | 1924 | 2006 | m | Chemistry, Biochemistry | Emigration | 1944 | Slowakia |
| **56** | Schild, Heinz Otto | 1906 | 1984 | m | Medicine | Emigration | 1932 | GB |
| **57** | Schlesinger, Max (alias Slazenger, ab 1939) | 1903 | 1971 | m | Medicine, Political Science | Emigration | 1933 | Burma/Myanmar |
| **58** | Schlossmann, Hans | 1894 | 1956 | m | Medicine | Emigration | 1935 | GB |
| **59** | Schnitzer, Robert Julius | 1894 | 1987 | m | Medicine | Emigration | 1939 | Canada |
| **60** | Slotta, Karl Heinrich | 1895 | 1987 | m | Chemistry | Emigration | 1935 | USA |
| **61** | Starkenstein, Emil | 1884 | 1942 | m | Medicine | Murder in Concentration Camp (1942) | 1939 | Netherlands |
| **62** | Sulman, Felix Gad | 1907 | 1986 | m | Veterinary Medicine, Human Medicine | Emigration | 1933 | Palestine |
| **63** | Taubmann, Gert | 1900 | 1983 | m | Medicine | Stayed in Germany | / |  |
| **64** | Unna, Klaus Robert Walter | 1908 | 1987 | m | Medicine | Emigration | 1933 | USA |
| **65** | Vogt, Marthe Louise | 1903 | 2003 | f | Medicine, Chemistry | Emigration | 1935 | GB |
| **66** | Waelsch, Heinrich Benedict (alias Waelsh) | 1905 | 1966 | m | Medicine | Emigration | 1938 | USA |
| **67** | Wasicky, Richard Balthasar | 1884 | 1970 | m | Medicine, Pharmacy | Emigration | 1938 | Brazil |
| **68** | Wilbrandt, Walther | 1907 | 1979 | m | Medicine | Emigration | 1934 | Switzerland |
| **69** | Wolff, Paul O. | 1894 | 1957 | m | Medicine | Emigration | 1933 | Switzerland |
| **70** | Wollenberger, Albert | 1912 | 2000 | m | Medicine, Biology | Emigration | 1933 | USA |
| **71** | Zak, Emil Rudolf | 1877 | 1949 | m | Medicine | Emigration | 1939 | USA |

**Table S1.** Data in this table were extracted from the German-language book of Löffelholz and Trendelenburg (2008) on persecuted German pharmacologists. M, male; f, female. The pharmacologists tabulated here constitute the basis for the analyses performed
